# Supplementary material for: Intended versus actual delivery location and factors associated with change in delivery location among pregnant women in Southern Province, Zambia: a prespecified secondary observational analysis of the ZamCAT
Source: BMJ Open. 2022 Mar 7;12(3):e055288. doi: 10.1136/bmjopen-2021-055288 (PMC8905985; doi:10.1136/bmjopen-2021-055288)
Supplement: Author's manuscript [file bmjopen-2021-055288.draft_revisions.pdf]

BMJ Open is committed to open peer review. As part of this commitment we make the peer review history of every article we publish publicly available.

When an article is published we post the peer reviewers' comments and the authors' responses online. We also post the versions of the paper that were used during peer review. These are the versions that the peer review comments apply to.

The versions of the paper that follow are the versions that were submitted during the peer review process. They are not the versions of record or the final published versions. They should not be cited or distributed as the published version of this manuscript.

BMJ Open is an open access journal and the full, final, typeset and author-corrected version of record of the manuscript is available on our site with no access controls, subscription charges or pay-per-view fees (http://bmjopen.bmj.com).

If you have any questions on BMJ Open's open peer review process please email info.bmjopen@bmj.com

BMJ Open

Where to deliver? Intention versus actual practice in pregnant women in Southern Province, Zambia

Journal:	*BMJ Open*
Manuscript ID	bmjopen-2021-055288
Article Type:	Original research
Date Submitted by the Author:	16-Jul-2021
Complete List of Authors:	Solomon, Hiwote; Boston University School of Public Health, Doctor of Public Health Program<br>Henry, Elizabeth; Harvard University T H Chan School of Public Health, Department of Global Health and Population; Boston University School of Public Health, Department of Global Health<br>Herlihy, Julie; Boston University School of Medicine, Pediatrics<br>Yeboah-Antwi, Kojo; Fr. Thomas Alan Rooney Memorial Hospital<br>Biemba, Godfrey; Boston University School of Public Health; Zambia National Health Research Authority<br>Musokotwane, Kebby; Levy Mwanawasa Medical University<br>Bhutta, Afsah ; Dow University of Health Sciences<br>Hamer, D.; Boston University School of Public Health, Department of Global Health; Boston University School of Medicine, Department of Medicine<br>Semrau, Katherine; Brigham and Women's Hospital, Ariadne Labs; Brigham and Women's Hospital, Division of Global Health Equity & Department of Medicine
Keywords:	PUBLIC HEALTH, Maternal medicine < OBSTETRICS, EPIDEMIOLOGY

SCHOLARONE™
Manuscripts

I, the Submitting Author has the right to grant and does grant on behalf of all authors of the Work (as defined in the below author licence), an exclusive licence and/or a non-exclusive licence for contributions from authors who are: i) UK Crown employees; ii) where BMJ has agreed a CC-BY licence shall apply, and/or iii) in accordance with the terms applicable for US Federal Government officers or employees acting as part of their official duties; on a worldwide, perpetual, irrevocable, royalty-free basis to BMJ Publishing Group Ltd ("BMJ") its licensees and where the relevant Journal is co-owned by BMJ to the co-owners of the Journal, to publish the Work in this journal and any other BMJ products and to exploit all rights, as set out in our licence.

The Submitting Author accepts and understands that any supply made under these terms is made by BMJ to the Submitting Author unless you are acting as an employee on behalf of your employer or a postgraduate student of an affiliated institution which is paying any applicable article publishing charge ("APC") for Open Access articles. Where the Submitting Author wishes to make the Work available on an Open Access basis (and intends to pay the relevant APC), the terms of reuse of such Open Access shall be governed by a Creative Commons licence – details of these licences and which Creative Commons licence will apply to this Work are set out in our licence referred to above.

Other than as permitted in any relevant BMJ Author's Self Archiving Policies, I confirm this Work has not been accepted for publication elsewhere, is not being considered for publication elsewhere and does not duplicate material already published. I confirm all authors consent to publication of this Work and authorise the granting of this licence.

For peer review only

Where to deliver? Intention versus actual practice in pregnant women in Southern Province, Zambia

Hiwote Solomon[1], Elizabeth G Henry[2,3], Julie M Herlihy[4], Kojo Yeboah-Antwi[5], Godfrey Biemba[1,6], Kebby Musokotwane[7], Afsah Bhutta[8], Davidson H. Hamer[3,9], Katherine E.A. Semrau[10,11,12]

[1] Boston University School of Public Health, Boston, Massachusetts, USA
[2] Department of Global Health and Population, Harvard TH Chan School of Public Health, Boston, Massachusetts, USA
[3] Department of Global Health, Boston University School of Public Health, Boston, Massachusetts, USA
[4] Department of Pediatrics, Boston University School of Medicine, Boston, Massachusetts, USA
[5] Fr. Thomas Alan Rooney Memorial Hospital, Asankrangwa, Western Region, Ghana
[6] Zambia National Health Research Authority, Lusaka, Zambia
[7] Levy Mwanawasa University Teaching Hospital, Lusaka, Zambia
[8] Dow University of Health Sciences, Karachi, Pakistan1 Section of Infectious Diseases,
[9] Department of Medicine, Boston University School of Medicine, Boston, MA, USA
[10] Ariadne Labs, Brigham and Women's Hospital and Harvard TH Chan School of Public Health, Boston, Massachusetts, USA
[11] Division of Global Health Equity & Department of Medicine, Brigham and Women's Hospital, Boston, Massachusetts, USA
[12] Department of Medicine, Harvard Medical School, Boston, Massachusetts, USA

Corresponding author
Hiwote Solomon, MPH
Boston University School of Public Health
801 Massachusetts Avenue
Boston, MA 02119
solomohi@bu.edu

For peer review only

Abstract

Objectives: The aim of this pre-specified, secondary analysis of the Zambia Chlorhexidine Application Trial (ZamCAT) was to determine the proportion of women who did not deliver where they had intended, to understand the underlying reasons for the discordance between planned and actual delivery locations; and to determine sociodemographic characteristics associated with where pregnant women intended to delivered [i.e. concordance with intention and practice].

Setting: Recruitment took place at 90 health facilities (HFs) in Southern Province, Zambia

Participants: A total of 39,679 pregnant women were enrolled in ZamCAT between Feb 15, 2011 and Jan 30, 2013.

Secondary outcome measures: The location where mothers gave birth (home vs. a HF) will be compared to their planned delivery location.

Results: When interviewed antepartum, 92% of respondents intended to deliver at a HF, 6.1% at home, and 1.2% had no plan. However, of those who intended to deliver at a HF, 61% did; of those who intended to deliver at home, only 4% did; and of those who intended to deliver at home, 2% delivered instead at a HF. Among women who delivered at home, we found that women who were aged 25-34 and $\geq$35 years were more likely to deliver where they intended than women aged 20-24 years (aOR=1.31, (95% CI=1.14, 1.50), p=0.17) and (aOR=1.32, (95% CI=1.12, 1.57), p=0.003), respectively. Women who delivered at HFs had greater odds of delivering where they intended if they received any primary schooling (aOR=1.34, (95% CI=1.09, 1.72), p=0.008) or more than a primary school education (aOR=1.54, (95% CI=1.17, 2.02), p=0.002), were literate (aOR=1.30 (95% CI=1.09, 1.54), p=0.003), and were not in the lowest quintile of the wealth index.

Conclusion: Understanding factors that influence women to change their birth plan as well as addressing structural and perceived barriers to facility delivery are important to improving care.

For peer review only

Article Summary

Strengths and limitations of this study:

- In this large, cluster-randomized controlled trial with more than 39,000 pregnant women enrolled, less than 4% of enrolled women were lost before delivery, and less than 1% were lost to follow-up during the neonatal period.
- The majority of women delivered in a facility (65%), but 35% delivered at home (or elsewhere). Women's intended delivery and actual delivery location were concordant for 67% of women; and discordant for 33%.
- Discrepancies found between intended and actual delivery location highlight that the development of birth plans and exposure to birth planning messaging are insufficient to address barriers to adhering to plans to have a facility-based childbirth.
- Social desirability bias among respondents as questions such as birth plan and reasons behind delivery choice may have influenced women to respond they intended to deliver in a facility
- Lack of qualitative data limited exploring the underlying motivations around intention and actual delivery locations

For peer review only

For peer review only - http://bmjopen.bmj.com/site/about/guidelines.xhtml

Introduction

Each year, 140 million newborns are delivered worldwide.[1] While there has been global progress in reducing child mortality, corresponding rates of reduction in newborn mortality have been far slower and annually 2.5 million newborn deaths occur.[2] As the world strives to meet the Sustainable Development Goals (SDGs) by 2030, it is important to understand not only the direct causes, but also health systems barriers and social determinants of health that lead to poor maternal and newborn outcomes.[3] Factors responsible for these outcomes include poverty, illiteracy, issues of women's empowerment and systematic marginalization of communities on the basis of gender, ethnicity or geography.[2,4] In sub-Saharan Africa, birth planning and facility-based delivery have been core components of interventions to improve maternal and neonatal outcomes. However, this has been insufficient as health systems need to be structurally redesigned to respond to local contexts.[5] Given the emphasis on birth planning in developing countries, it is important to understand why women deliver in locations that differ from what they intended, and what contributes to that change in plan, in order to adequately design interventions and messaging to address specific barriers that prevent women from delivering where they intended.

Health facility delivery at primary health facilities with support from referral-level facilities for complicated births has been shown to improve maternal and neonatal outcomes.[5–9] In order to better understand some of the underlying factors and determinants of access to health facilities equipped with emergency obstetrical and newborn care (EmONC) and skilled birth attendants in rural Zambia, we used data from the Zambia Chlorhexidine Application Trial (ZamCAT), a cluster randomized controlled trial that assessed whether daily 4% chlorohexidine cord cleansing was more effective than dry cord care for prevention of neonatal deaths and omphalitis (umbilical cord

For peer review only - http://bmjopen.bmj.com/site/about/guidelines.xhtml

infection) in Southern Province.[10] The aim of this pre-specified, secondary analysis was to determine the proportion of women who did not deliver where they had intended, to understand the underlying reasons for the discordance between planned and actual delivery locations; and to determine sociodemographic characteristics associated with where pregnant women intended to delivered [i.e. concordance with intention and practice].

Methods

Study Site:

Southern Province, one of ten provinces in Zambia, consists of 13 districts with an estimated population of 2.1 million in 2020.[11] At the time of the study, the 2013-14 Zambia Demographic Health Survey (ZDHS) reported that nearly all women (96%) received at least one antenatal care visit during pregnancy.[12] Additionally, more than half of women (55.9%) in Southern Province delivered in a health facility (predominately public sector facilities), while 41% delivered at home.[12] Skilled birth attendants were present for 55% of all deliveries in Southern Province, while untrained family or relatives assisted with 22% of home deliveries.[12]

Study Design

ZamCAT, conducted from 2011-2013, was a cluster-randomized trial that enrolled and followed a cohort of 39,679 pregnant women living in six districts of Southern Province from before delivery to 28 days after delivery (ClinicalTrials.gov Identifier: NCT01241318). The analysis presented here was an *a priori* research question focusing on the secondary outcomes of the ZamCAT study: where did pregnant women enrolled in ZamCAT plan to deliver, what were their

For peer review only - http://bmjopen.bmj.com/site/about/guidelines.xhtml

reasons for their intended location, and what was their actual practice? Our analysis also assessed factors that may have influenced intended and actual delivery locations.

In the main trial, the units of randomization (clusters) for ZamCAT were Zambian government-run or mission-run primary health-care centers in Southern Province. Ninety clusters were randomized into two groups: (1) clean dry cord care in accordance with Ministry of Health guidelines (control) and (2) daily 4% chlorhexidine cord washes until three days after the umbilical cord fell off (intervention). The study location included a wide range of health facilities, both urban and rural, with differences in staffing and catchment area population characteristics.

Eligible women were in their second or third trimester, aged 15 years or older, planning to stay in the catchment area until 28 days postpartum, and willing to provide informed consent and complete cord care as per cluster assignment.[10] Women were screened by field monitors (data collectors), and women were enrolled either at a facility-based antenatal care visit or through community outreach events. At enrollment, a detailed questionnaire was administered which included questions on household information, birth plan, and antenatal care characteristics. After the enrollment survey was completed, participants were encouraged to deliver at the nearest health facility and were given standard newborn care messages which included information about delivery location, breastfeeding, cord care and danger signs of ill health in their newborn baby as per national and Ministry of Health guidelines. At a home visit two weeks after enrollment, all participants, irrespective of study allocation, were provided a standard clean delivery kit. A birth notification system was developed in collaboration with the community specific to each cluster to alert study team of births.[13] For both study arms, field monitors conducted routine home visits at

For peer review only - http://bmjopen.bmj.com/site/about/guidelines.xhtml

least once during the antenatal period and four times after delivery (days 1, 4, 10, and 28 postpartum). Detailed information on where the woman delivered and why was collected during the day 4 postpartum visit. Data were collected using paper forms designed in the Teleforms system (HP Cambridge, UK). Supervisors reviewed forms for illegible and missing entries and worked with the field monitors to make sure they were complete. The forms were transported to the central study office in Choma where a data management team scanned and entered them into a Microsoft Access Database.

Patient and Public Involvement

Study preparations required baseline formative ethnographic research, substantial community sensitization, and engagement with three levels of stakeholders, each necessitating different strategies. The process of community engagement for ZamCAT is detailed further by Hamer et al.[13] Community leaders and neighborhood health committees in the 90 clusters identified appropriate Field Monitor (data collector) candidates according to specific selection criteria. The local health facility staff assisted by ranking lists of potential data collectors provided by the community. Further, the effective birth notification system was developed by the community leaders. Results from the primary study were shared through community meetings in collaboration with the district health offices and at the Zambian National Health Research Conference.

Statistical analysis

Using the full cohort of women enrolled in the study, we assessed women's socioeconomic and pregnancy-related characteristics and related them to the outcome of interest (delivery where

For peer review only - http://bmjopen.bmj.com/site/about/guidelines.xhtml

intended). Our primary outcome was a four-level categorical variable: intended to deliver at health facility and did, intended to deliver at home and did, intended to deliver at a health facility but delivered at home, and intended to deliver at home but delivered at a health facility. Socioeconomic and pregnancy-related characteristics were documented during the enrollment visit and compared across the intention/practice groups. Pregnancy-related characteristics potentially associated with the outcomes were compared between the two groups using the $\chi 2$ test, Fisher's exact test, t-tests, or non-parametric Wilcoxon rank sum tests, as appropriate for continuous or categorical variables. We also assessed the reasons women indicated for why they intended to deliver at a certain location and reasons given after actual delivery on where they actually delivered. All statistical analyses were completed using SAS University Edition®.[14] We assessed the association of sociodemographic characteristics with women delivering where they intended to deliver or elsewhere using logistic regression modeling. We also conducted a stratified logistic regression model to assess the predictors of women delivering where they intended stratified by actual delivery location (home and facility).

The Boston University Medical Campus Institutional Review Board (FWA# 00008404) and University of Zambia Research Ethics Committee (FWA# 00001131) provided ethical clearance. The Zambia National Health Research Authority approved the study. All women provided written informed consent, which was obtained in English or Tonga.

Results

A total of 39,679 pregnant women were enrolled in ZamCAT between Feb 15, 2011 and Jan 30, 2013.[10] During the study 38,131 women had 38,579 deliveries; 1,548 enrolled women were

For peer review only - http://bmjopen.bmj.com/site/about/guidelines.xhtml

excluded as a result of false pregnancies, miscarriages or abortion, withdrawal from study before delivery, lost to follow-up, or death. We had intended and actual delivery information on 37,105 of the enrolled population. The mean age (± standard deviation [SD]) of the women was 25.6 ± 6.9 years, 10% had no formal education, and 52% had only a primary education (Table 1). Of the 37,105 with birth plans stated, 467 (1.2%) had no plan, 2,278 (6.1%) planned to deliver at home, 33,953 (91.5%) intended to deliver at a health facility, and 407 (1.1%) stated other. In practice, 13,139 (35.4%) actually delivered at home, 23,666 (63.7%) actually delivered at a health facility, and 300 (0.8%) delivered in another location.

Table 1. Demographic, household, and pregnancy characteristics of women enrolled in ZamCAT

**Characteristics**	Intended to deliver at health facility and did **n=22 651**	Intended to deliver at home and did **n=1515**	Intended to deliver at health facility but delivered at home **n=11 016**	Intended to deliver at home but delivered at a health facility **n=755**
Maternal Age (years), n (%)				
<20	5919 (26.1%)	185 (12.2%)	2115(19.2%)	215 (28.5%)
20-35	14473 (63.9%)	1114 (73.5%)	7626 (69.2%)	442 (58.5%)
>35	2259 (10.0%)	216 (14.3%)	1275 (11.6%)	98 (13.0%)
Missing				
Tribe, n (%)				
Tonga	19 295 (85.2%)	1431 (94.5%)	10 257 (93.1%)	670 (88.7%)
Ila	142 (0.6%)	7 (0.5%)	53 (0.5%)	4 (0.5%)
Lozi	1175 (5.2%)	20 (1.3%)	238 (2.2%)	28 (3.7%)
Nyanja	687 (3.0%)	18 (1.2%)	147 (1.3%)	13 (1.7%)
Bemba	568 (2.5%)	23 (1.5%)	135 (1.2%)	24 (3.2%)
Other	781 (3.4%)	15 (1.0%)	183 (1.7%)	12 (1.6%)
Missing	3 (0.08%)	1 (0.1%)	3 (0.03%)	4 (0.5%)
Women's Education Level, n (%)				
No education	1681 (7.4%)	265 (17.5%)	1442 (13.1%)	95 (12.6%)
Some primary education	10 779 (47.6%)	1001 (66.1%)	6487 (58.9%)	366 (48.5%)
Some secondary education	9884 (43.6%)	246 (16.2%)	3067 (27.8%)	290 (38.4%)
Higher than secondary education	288 (1.3%)	2 (0.13%)	14 (0.1%)	0 (0.0%)
Unknown or Missing	19 (0.1%)	1 (0.1%)	6 (0.06%)	4 (0.5%)
Maternal Literacy, n (%)				
Illiterate	4875 (21.5%)	611 (40.3%)	3720 (33.8%)	226 (29.9%)
Can read a bit	10620 (46.9%)	716 (47.3%)	5212 (47.4%)	332 (44.0%)
Can read very well	7078 (31.2%)	177 (11.7%)	2022 (18.4%)	191 (25.3%)
No response or Missing	78 (0.3%)	11 (0.7%)	62 (0.6%)	6 (0.8%)
Wealth Index, n (%)				
1-lowest	5214 (23.0%)	541 (35.7%)	3154 (28.6%)	246 (32.6%)
2	5407 (23.9%)	421 (27.8%)	2910 (26.4%)	175 (23.2%)

For peer review only - http://bmjopen.bmj.com/site/about/guidelines.xhtml

3	5490 (24.2%)	361 (23.8%)	2807 (25.5%)	181 (24.0%)
4-highest	6540 (28.9%)	192 (12.7%)	2145 (19.5%)	151 (20.0%)
Marital Status, n (%)				
Single	4173 (18.4%)	82 (5.4%)	1243 (11.3%)	132 (17.5%)
Married or Cohabitating	18 267 (80.7%)	1405 (92.7%)	9644 (87.6%)	600 (79.5%)
Separated, Divorced, or Widowed	204 (0.9%)	26 (1.7%)	128 (1.2%)	19 (2.5%)
Missing	7 (0.03%)	2 (0.1%)	1 (0.01%)	4 (0.5%)
Median household size (IQR)	6 (4-8)	6 (4-8)	6 (4-8)	6 (4-8)
Time walking from home to health facility				
<1h	9276 (41.0%)	293 (19.3%)	2301 (20.9%)	253 (33.5%)
1 to <2 h	7823 (34.5%)	526 (34.7%)	4202 (38.1%)	278 (36.8%)
2 to <3h	3981 (17.6%)	449 (29.6%)	3054 (27.7%)	140 (18.5%)
3 to <4 h	1047 (4.6%)	167 (11.0%)	1017 (9.2%)	58 (7.7%)
4 to <5 h	250 (1.1%)	43 (2.8%)	248 (2.3%)	12 (1.6%)
5 h or more	120 (0.5%)	12 (0.8%)	129 (1.2%)	8 (1.1%)
Unknown	81 (0.4%)	17 (1.1%)	42 (0.4%)	2 (0.3%)
Missing	74 (0.3%)	8 (0.5%)	23 (0.2%)	4 (0.5%)
**Pregnancy Characteristics**				
Parity, mean (SD)	2.7 (2.3)	2.4 (2.3)	2.3 (2.3)	2.5 (2.3)
Primiparous, n (%)	4230 (18.7%)	231 (15.3%)	2188 (19.9%)	119 (15.8%)
Multiparous, n (%)	11 939 (52.7%)	1131 (74.7%)	7086 (64.3%)	401 (53.1%)
Missing, n (%)	6482 (28.6%)	153 (10.1%)	1742 (15.8%)	235 (31.1%)
Gravida, mean (SD)	3.8 (2.4)	3.5 (2.3)	3.4 (2.3)	3.5 (2.3)
Primigravida, n (%)	6327 (27.9%)	148 (9.8%)	1702 (15.5%)	225 (29.8%)
Multigravida, n (%)	16 294 (71.4%)	1362 (89.9%)	9298 (84.4%)	525 (69.5%)
Missing, n (%)	30 (0.1%)	5 (0.3%)	16 (0.2%)	5 (0.7%)
Gestational age at enrollment, mean (SD)	27.9 (6.8)	28.0 (7.0)	28.1 (7.1)	28.1 (7.0)
Time between water breaking and delivery, n (%)				
Less than 24 hours	20,259 (91.7%)	1344 (88.7%)	10,123 (91.9%)	418 (55.4)
24-48 hours	781 (3.5%)	25 (1.7%)	168 (1.5%)	35 (4.6%)
More than 48 hours	120 (0.54%)	8 (0.53%)	26 (0.2%)	2 (0.3%)
Don't know	915 (4.1%)	120 (8.0%)	446 (4.1%)	32 (4.2%)
Missing	576 (2.5%)	1 (1.2%)	253 (2.3%)	268 (35.5%)

*Those who responded to having "no plan" or "other" when asked about intended delivery location, and those who responded as "other" when asked about actual delivery location were excluded (n=1,168).

Reasons for planned vs. actual delivery location

At study enrollment, respondents were asked about their birth plan and where they planned to deliver as well as why (Table 2). Almost all women had a delivery location in their plan (98.8%) and nearly all women (91.6%) reported that they planned to deliver in a health facility. Women who planned to deliver in a health facility stated they were motivated by safety for mother/baby

(83.9%) and need for skilled attendance (81.3%). Among those who intended to deliver at home, distance was the most commonly noted reason (37.9%) and same location as previous deliveries (29.2%).

Table 2. Planned delivery location and rationale

	No Plan (N=467)		Health Facility (N=33,953)		Home (N=2,278)		Other (N=407)	
	n	%	n	%	n	%	n	%
Same location as prior delivery	3	0.6%	4,237	12.5%	666	29.2%	55	13.5%
Need for skilled attendance	14	3.0%	27,578	81.2%	402	17.7%	281	69.0%
Financial restraints	13	2.8%	1,058	3.1%	443	19.5%	16	3.9%
Distance	10	2.1%	4,714	13.9%	851	37.4%	45	11.1%
Relationship with Providers	3	0.8%	453	1.3%	175	7.7%	8	2.0%
Family/social expectations	2	0.5%	714	2.1%	264	11.6%	2	0.5%
Safety for mother/baby	12	2.6%	28,548	84.1%	464	20.4%	293	72.0%
Other	3	0.6%	276	0.8%	47	2.1%	33	8.1%
Missing	0	0.0%	1	0.0%	5	0.2%	4	1.0%

**Note "Other" in birth plan often includes HOSPITAL delivery which is separate from health facility delivery as well as TBA delivery.*

After delivery, women were asked about where they actually delivered and why (Table 3). Although nearly all women indicated that they planned to deliver at a health facility, only 63.8% (N=23,666) actually did. The primary motivations for delivery in the facility was need for skilled attendance (54.6%) and safety for the mother and baby (55.1%). While only 6% of women intended to deliver at home, over one-third (35.4%) of women actually did (N=13,139). The top three reasons for delivering at home were distance (29.7%), need for skilled attendance (43.0%), and safety for mother/baby (44.7%).

Table 3. Actual delivery location and reasoning

	Health Facility (N=23,666)		Home (N=13,139)		Other (N=300)	
	n	%	n	%	n	%
Same location as prior delivery	3,182	13.5%	2,062	15.7%	47	15.7%
Need for skilled attendance	12,916	54.6%	5,654	43.0%	164	54.7%
Financial restraints	1799	7.6%	2,234	9.4%	13	4.3%

For peer review only - http://bmjopen.bmj.com/site/about/guidelines.xhtml

Distance	6,303	26.6%	3,898	29.7%	74	24.7%
Relationship with Providers	571	2.4%	426	3.2%	9	3.0%
Family/social expectations	1,251	5.3%	961	7.5%	17	5.7%
Safety for mother/baby	13,048	55.1%	5,866	44.7%	170	56.7%
Other	1,468	6.2%	792	6.0%	23	7.7%
Missing	1,353	5.7%	762	5.8%	15	5.0%

Note: Health facility in actual delivery location includes hospital delivery

Intended vs. actual delivery location

Overall, 67% of women delivered in their intended location. Almost two-thirds of women (n=22,651, 61.1%) who intended to deliver at a health facility actually did (concordant); in contrast, almost one-third of women who intended to deliver at a health facility (n=11,016, 29.7%) delivered at home (discordant). Of those who intended to deliver at home, only 4.1% (n=1515) actually did. Additionally, of those women who intended to deliver at home, 2.0% (n=755) delivered instead at a health facility. Excluded were those who indicated "no plan" or "other" for intended delivery location and those who indicated "other" for actual delivery location.

We observed that 18.7% of those who intended to deliver at a heath facility and did were primiparous, compared to women who intended to deliver at a health facility but delivered at home (19.9%). For those women who were multiparous, more than half (52.7%) intended to deliver at home and actually did; two-thirds (64.3%) of women who intended to deliver at a health facility, delivered at home. We found that time between water breaking and delivering in less than 24 hours was not shorter for those who intended to delivery at a health facility but delivered at home. Women who intended to deliver at home and subsequently delivered at a health center stated they needed a skilled attendant for the safety of the mother and baby (Table 4). About 30% of respondents, who indicated distance as a reason for delivering where they did, delivered at home. For those who intended to deliver at a health facility and did, 41% lived less than a one hour walk

For peer review only

For peer review only - http://bmjopen.bmj.com/site/about/guidelines.xhtml

from the health facility. Of women who intended to deliver at home and did, 64% lived between one to three hours walk from the nearest health facility. Among those who intended to deliver at a health facility and actually delivered at home, 46.3% indicated that they did so because of safety for mother/baby. Additionally, this same group indicated a need for skilled attendance as a reason for delivering at home versus at a health facility. Among those who intended to and actually delivered at home, only 8.9% indicated family/social expectations as a reason while 34.5% indicated safety for mother/baby as a reason.

Table 4. Comparison of Intended and Actual Delivery Location, with Reasons for Actual Delivery Location

Reasons for Actual Delivery Location	Intended to deliver at health facility and did (N=22 651)		Intended to deliver at home and did (N=1515)		Intended to deliver at health facility but delivered at home (N=11 016)		Intended to deliver at home but delivered at a health facility (N=755)	
	**n**	**%**	**n**	**%**	**n**	**%**	**n**	**%**
Same location as prior delivery	3020	13.4%	279	18.4%	1677	15.2%	120	15.9%
Need for skilled attendance	12458	54.5%	476	31.4%	4947	44.9%	353	46.8%
Financial restraints	1708	7.7%	149	9.8%	1021	9.3%	72	9.5%
Distance	5987	26.6%	505	33.3%	3229	29.3%	233	30.9%
Relationship with Providers	528	2.4%	76	5.0%	326	3.0%	34	4.5%
Family/social expectations	1182	5.3%	135	8.9%	782	7.1%	48	6.4%
Safety for mother/baby	12585	36.9%	523	34.5%	5095	46.3%	332	44.0%
Other	1408	6.2%	107	7.1%	654	5.9%	44	5.8%
Missing	1296	6.2%	89	5.9%	628	5.7%	47	6.2%
**Time walking from home to health facility**								
<1h	9276	41.0%	293	19.3%	2301	20.9%	253	33.5%
1 to <2 h	7823	34.5%	526	34.7%	4202	38.1%	278	36.8%
2 to <3h	3981	17.6%	449	29.6%	3054	27.7%	140	18.5%
3 to <4 h	1047	4.6%	167	11.0%	1017	9.2%	58	7.7%
4 to <5 h	250	1.1%	43	2.8%	248	2.3%	12	1.6%
5 h or more	120	0.5%	12	0.8%	129	1.2%	8	1.1%
Unknown	81	0.4%	17	1.1%	42	0.4%	2	0.3%
Missing	74	0.3%	8	0.5%	23	0.2%	4	0.5%

For peer review only - http://bmjopen.bmj.com/site/about/guidelines.xhtml

Predictors of women delivering where intended

Table 5 shows the result of our stratified logistic regression model where we assessed predictors of women delivering where they intended to, stratified by home delivery and facility delivery. Among women who delivered at home, we found that women who were aged 25-34 and ≥ 35 years were more likely to deliver where they intended than women aged 20-24 years (aOR=1.31, (95% CI=1.14, 1.50), p=0.17) and (aOR=1.32, (95% CI=1.12, 1.57), p=0.003), respectively. Women who were 15-19 years and delivered at home had lower odds (aOR=0.77, (95% CI=0.60, 0.93), p<.0001) of delivering where they intended compared to those aged 20-24 years. Women who delivered at home and had more than a primary education were less likely to deliver where they intended when compared to those with no education (aOR=0.55, (95% CI=0.43, 0.69), p<0.0001). Those who delivered at home and were not in the lowest wealth quintile were less likely to deliver where they intended (Table 5). When examining the time to walk to nearest health facility, we found that those who delivered at home had higher odds of delivering where they intended to if they lived two to less than three hours from nearest health facility (aOR=1.16, (95% CI=1.02, 1.33), p=0.03) or three to less than four hours away from nearest health facility (aOR=1.27, (95% CI=1.05, 1.54), p=0.01).

Women who delivered at the facility had greater odds of delivering where they intended if they received any primary schooling (aOR=1.34, (95% CI=1.09, 1.72), p=0.008) or more than a primary school education (aOR=1.54, (95% CI=1.17, 2.02), p=0.002), were literate (aOR=1.30 (95% CI=1.09, 1.54), p=0.003), were in the second (aOR=1.37, (95% CI=1.16, 1.63), p=0.0003), third (aOR=1.34, (95% CI: (1.13, 1.58), p=0.0007), or fourth (aOR=1.69, (95% CI=1.40, 2.04),

For peer review only - http://bmjopen.bmj.com/site/about/guidelines.xhtml

p<0.0001) quintile of the wealth index, and those who experienced headache as a complication during pregnancy (aOR=1.71, (95% CI=1.12, 2.60), p=0.01). In contrast, women who delivered at health facility had lower odds of delivering where they intended if it was their first pregnancy (aOR=0.67, (95% CI=0.55, 0.80), p<0.0001) compared to those who had been pregnant before, lived between a three to less than four hour walk from the nearest health facility (aOR=0.74, (95% CI=0.57, 0.96), p=0.02) compared to those who lived a one to less than two hour walk, lapsed 24-48 hours between water breaking and delivery (aOR=0.534 (95% CI=0.40, 0.73), p<0.0001) compared to those who only lapsed less than 24 hours, and those who experienced bleeding (aOR=0.60, (95% CI=0.40, 0.90), p=0.01), fits (aOR=0.40, (95% CI=0.29, 0.85), p=0.02), vaginal discharge (aOR=0.24, (95% CI=0.13, 0.42), p<.0001), and/or heavy bleeding (aOR=0.73, (95% CI=0.56, 0.97), p=0.03) compared to those who did not experience these symptoms.

Table 5. Predictors of women delivering where they intended to, stratified by delivery location

	**Among those that delivered at home, adjusted odds ratio (aOR) of delivering where intended**		**Among those that delivered at facility, adjusted odds ratio (aOR) of delivering where intended**	
**Characteristics**	**aOR (95% CI)**	**p-value**	**aOR (95% CI)**	**p-value**
Maternal Age (years)				
15-19	**0.77 (0.64 0.93)**	**0.007**	**0.69 (0.57, 0.82)**	**<.0001**
20-24	ref		ref	
25-34	**1.31 (1.14, 1.50)**	**<.0001**	0.89 (0.75, 1.05)	**0.17**
>35	**1.32 (1.12, 1.57)**	**0.001**	**0.73 (0.59, 0.90)**	**0.003**
Women's Education Level				
No education	ref		ref	
Any primary	0.90 (0.75, 1.06)	0.21	**1.34 (1.09, 1.72)**	**0.008**
More than primary	**0.55 (0.43, 0.69)**	**<.0001**	**1.54 (1.17, 2.02)**	**0.002**
Marital Status				
Married	ref		ref	
Not married	**0.67 (0.55, 0.83)**	**0.0002**	0.89 (0.76, 1.06)	0.18
Can read				
No	ref		ref	
Yes	1.05 (0.91, 1.20)	0.50	**1.30 (1.09, 1.54)**	**0.003**
Household Light Source				
No electricity	ref		ref	
Electricity	0.81 (0.57, 1.16)	0.25	1.27 (0.95, 1.69)	0.11
Wealth Index				

For peer review only - http://bmjopen.bmj.com/site/about/guidelines.xhtml

1-lowest	**ref**	**ref**	**ref**	**ref**
2	**0.85 (0.74, 0.97)**	**0.02**	**1.37 (1.16, 1.63)**	**0.0003**
3	**0.76 (0.66, 0.88)**	**0.0002**	**1.34 (1.13, 1.58)**	**0.0007**
4-highest	**0.56 (0.46, 0.67)**	**<.0001**	**1.69 (1.40, 2.04)**	**<0.0001**
Primigravida				
No	ref		ref	
Yes	0.90 (0.73, 1.11)	0.33	**0.67 (0.55, 0.80)**	**<0.0001**
Time to walk to nearest health facility				
<1h	1.10 (0.94, 1.28)	0.24	1.14 (0.98, 1.32)	0.10
1 to <2 h	ref		ref	ref
2 to <3h	**1.16 (1.02, 1.33)**	**0.03**	0.92 (0.78, 1.09)	0.34
3 to <4 h	**1.27 (1.05, 1.54)**	**0.01**	**0.74 (0.57, 0.96)**	**0.02**
4 to <5 h	1.20 (0.84, 1.68)	0.32	0.80 (0.48, 1.35)	0.41
5 h or more	0.68 (0.37, 1.24)	0.20	0.70 (0.34, 1.45)	0.34
Time between water breaking and delivery				
Less than 24 hours	**ref**		ref	
24-48 hours	1.04 (0.68, 1.59)	0.87	**0.54 (0.40, 0.73)**	**<0.0001**
More than 48 hours	2.11 (0.95, 4.69)	0.07	1.34 (0.44, 4.38)	0.58
Don't know	**1.93 (1.56, 2.38)**	**<.0001**	**0.49 (0.37, 0.64)**	**<0.0001**
Complications during pregnancy*				
High fever	1.45 (1.00, 2.11)	0.05	0.79 (0.54, 1.16)	0.23
Bleeding	1.23 (0.80, 2.05)	0.31	**0.60 (0.40, 0.90)**	**0.01**
Fits	2.15 (0.79, 5.82)	0.13	**0.40 (0.19, 0.85)**	**0.02**
Headache	1.07 (0.79, 1.44)	0.67	**1.71 (1.12, 2.60)**	**0.01**
Vaginal discharge	0.50 (0.15, 1.61)	0.24	**0.24 (0.13, 0.42)**	**<0.0001**
Heavy bleeding	1.08 (0.78, 1.50)	0.63	**0.73 (0.56, 0.97)**	**0.03**
Swelling	0.88 (0.63, 1.22)	0.43	0.82 (0.62, 1.10)	0.19

* Note: Referent groups for complications were the absence of specified complications
**Adjusted for maternal age, women's education, marital status, literacy, wealth index, housing light source

Discussion

Overall, birth plans were widely prepared in this cohort and >90% of women indicated a plan to deliver at a health facility, consistent with the 2013-2014 Zambia Demographic Health Survey (ZDHS). [12] However, only two-thirds of women had actual delivery location in line with their birth plan and >35% of women delivered at home. Our analysis demonstrates clear deviations from the birth plan during the actual delivery, especially among those who had expressed an intent to deliver at a facility. Discrepancy between planning and actual delivery behavior highlights that the

For peer review only - http://bmjopen.bmj.com/site/about/guidelines.xhtml

development of birth plans and exposure to birth planning messaging are insufficient to address barriers to adhering to plans to have a facility-based childbirth.

Several reasons may underlie the discrepancy between intended location and actual practice around childbirth. One potential reason could be due to women's increased concerns about safety at facilities that are poorer quality. Interestingly, 46% of women in our sample indicated safety for mother/baby as a reason for why they delivered at home even though they intended to deliver at a health facility. Unfortunately, we do not know if this was driven by fear of poor treatment at the facility or fear of transporting/moving the laboring women. In some instances, poorly functioning health systems are unable to engage women in antenatal and post-natal care to create sufficient continuity of care.[15] Some of these barriers may relate to a sense of fatalism around maternal and newborn care, and are important contributors to preventable mortality.[16] Such disparities present a major challenge to policies and programs aiming to mitigate the burden of maternal and neonatal mortality. The large proportion of facility births in unclean, unhygienic environments, and poor postnatal care may be important contributors to why women indicate safety for mother/baby as a reason why they changed their birth plan from intending to deliver at a health facility to delivering at home.

Another factor that may contribute to discrepancy between intention and practice is the dynamic between provider and patient.[17–19] Among those who intended to deliver at a health facility but instead delivered at home, 45% indicated need for skilled delivery as a reason. While this seems counterintuitive, one reason for this may be the presence of traditional birth attendants (TBAs) in the community who women regard as skilled attendants.[20,21] Although a policy change in Zambia

For peer review only - http://bmjopen.bmj.com/site/about/guidelines.xhtml

resulted in TBAs no longer being trained and recommended that they stop conducting deliveries at home, a study on reasons for home delivery and use of TBAs in rural Zambia by Sialubanje et al. found that most women had positive attitudes towards TBAs and regarded them as respectful, skilled, and trustworthy.[17] Moreover, most women indicated that negative attitudes toward the quality of services at the clinic as a reason for why they delivered at home.[17] Smith et al. focused on behavioral drivers in labor and delivery wards in Chipata District in Eastern Province, Zambia and found that women in the wards are often subjected to disrespect and abuse.[22] Another study in rural Kenya found that although the community was aware of the risks of delivering with a TBA, many still preferred to deliver with a TBA due to their availability, accessibility, and their friendly attitude.[20] In Zambia, a study by Biemba et al. which utilized data from the ZamCAT study, found that TBAs often end up tending to deliveries even in health facilities due to a lack of qualified personnel.[23] As for the safety of mother and child, some women perceive delivering at the health facility as unsafe based on cultural values of elder women in the community.[24] Finally, in another study, women in Ethiopia who had low perceived susceptibility and severity were more likely to intend to deliver at home instead of at a health facility.[24,25]

Among women who intended to deliver at home distance was most often cited as a reason (37.9%), although this less often reported when women were asked why they delivered home post-delivery. Our data are similar to the 2013-14 ZDHS which reported that 32.2% of women in Southern Province indicated distance and lack of transportation as a reason for not delivering in a health facility. Additionally, a study in Ebonyi State, Nigeria on the choice of birth place among antenatal clinic attendees in rural mission hospitals also found that distance served as a major determinant of birth place location.[26] Distance as a barrier to accessing care is not uncommon in many resource-

For peer review only - http://bmjopen.bmj.com/site/about/guidelines.xhtml

constrained countries in sub-Saharan Africa and Asia.[27,28] Scott el al. found that women who lived in remote areas of Zambia faced persistent challenges regarding delivery location.[29] The study which included four districts in Southern Province also found women who lived far from a health facility, attended fewer antenatal visits, were multigravida, and who did not utilize maternity waiting homes had higher odds of home delivery.[29] Maternal waiting homes have shown to increase likelihood of delivering at a health facility.[29–31] Given that most maternal waiting homes were renovated or built after 2013, our study did not collect data on how maternal waiting home influenced delivery locations for those further away from the health facility.

Our analysis is strengthened by the sample size and variation in adherence to intended delivery location. However, a key limitation is a likelihood for social desirability bias among respondents as questions such as birth plan and reasons behind delivery choice may have influenced women to respond they intended to deliver in a facility. Further, we do not have qualitative data could have helped explore the underlying motivations around intention and actual delivery locations.

Understanding factors that influence women to change their birth plan as well as addressing structural and perceived barriers to facility delivery are important to improving care. Maternal and neonatal outcomes can be significantly improved if women delivered at a facility with at least basic emergency obstetric and newborn care.[32,33] Potential interventions that can address barriers to reaching facilities include provision of maternity waiting homes, addressing perceived safety and treatment of mothers and infants in facilities, supporting providers to provide high-quality/respectful maternity care, systemwide plans that support transport of pregnant women to a health facility, messaging around saving for transport to a health facility, further health

For peer review only - http://bmjopen.bmj.com/site/about/guidelines.xhtml

education for those who are primigravida to deliver a health facility, and safe delivery incentive programs.

Contributionship statement:

As the original co-investigators of the ZamCAT trial, DHH and KEAS were responsible for conceiving the original research question, designing the trial, and overseeing the study implementation. HS was responsible for conceptualization of the sub-analysis, methodology, conducting formal analysis, and the writing and revision of the manuscript. JMH, GB, KY-A were part of the original research team for ZamCAT and were responsible for conceptualization, methodology, and the reviewing and editing of the manuscript. AB was responsible for formal analysis and the reviewing and editing of the manuscript. KM was responsible for conceptualization, and the reviewing and editing of the manuscript.

Competing interests: None declared.

For peer review only

References

1. Graham W, Woodd S, Byass P, et al. Diversity and divergence: the dynamic burden of poor maternal health. *The Lancet*. 2016;388(10056):2164-2175.

2. UNICEF. Neonatal mortality. UNICEF DATA. Published 2018. Accessed May 13, 2020. https://data.unicef.org/topic/child-survival/neonatal-mortality/

3. United Nations. Transforming our world: the 2030 Agenda for Sustainable Development | Department of Economic and Social Affairs. Published 2015. Accessed January 9, 2021. https://sdgs.un.org/2030agenda

4. Liu L, Oza S, Hogan D, et al. Global, regional, and national causes of under-5 mortality in 2000–15: an updated systematic analysis with implications for the Sustainable Development Goals. *The Lancet*. 2016;388(10063):3027-3035.

5. Hanson C, Waiswa P, Pembe A, Sandall J, Schellenberg J. Health system redesign for equity in maternal and newborn health must be codesigned, country led, adapted to context and fit for purpose. *BMJ Glob Health*. 2020;5(10):e003748. doi:10.1136/bmjgh-2020-003748

6. Doctor HV, Nkhana-Salimu S, Abdulsalam-Anibilowo M. Health facility delivery in sub-Saharan Africa: successes, challenges, and implications for the 2030 development agenda. *BMC Public Health*. 2018;18(1):765.

7. Pervin J, Moran A, Rahman M, et al. Association of antenatal care with facility delivery and perinatal survival–a population-based study in Bangladesh. *BMC Pregnancy Childbirth*. 2012;12(1):111.

8. Campbell OMR, Graham WJ, Lancet Maternal Survival Series steering group. Strategies for reducing maternal mortality: getting on with what works. *Lancet Lond Engl*. 2006;368(9543):1284-1299. doi:10.1016/S0140-6736(06)69381-1

9. Roder-DeWan S, Nimako K, Twum-Danso NAY, Amatya A, Langer A, Kruk M. Health system redesign for maternal and newborn survival: rethinking care models to close the global equity gap. *BMJ Glob Health*. 2020;5(10):e002539. doi:10.1136/bmjgh-2020-002539

10. Semrau KEA, Herlihy J, Grogan C, et al. Effectiveness of 4% chlorhexidine umbilical cord care on neonatal mortality in Southern Province, Zambia (ZamCAT): a cluster-randomised controlled trial. *Lancet Glob Health*. 2016;4(11):e827-e836. doi:10.1016/S2214-109X(16)30215-7

11. Zambia Statistics Agency. Population and Demographic Projections, 2011-2035 - Zambia Data Portal. Published 2020. Accessed January 31, 2021. https://zambia.opendataforafrica.org/ZMPHC2015/population-and-demographic-projections-2011-2035

For peer review only - http://bmjopen.bmj.com/site/about/guidelines.xhtml

12. Central Statistical Office/Zambia, Ministry of Health/Zambia, University of Zambia Teaching Hospital Virology Laboratory, University of Zambia Department of Population Studies, Tropical Diseases Research Centre/Zambia, I. C. F. International. Zambia Demographic and Health Survey 2013-14. Published online March 1, 2015. Accessed February 14, 2020. https://dhsprogram.com/publications/publication-fr304-dhs-final-reports.cfm

13. Hamer DH, Herlihy JM, Musokotwane K, et al. Engagement of the community, traditional leaders, and public health system in the design and implementation of a large community-based, cluster-randomized trial of umbilical cord care in Zambia. *Am J Trop Med Hyg*. 2015;92(3):666-672.

14. SAS Institute. *SAS University Edition*.; 2020. Accessed January 9, 2021. https://support.sas.com/en/software/university-edition-support.html

15. Kyei-Nimakoh M, Carolan-Olah M, McCann TV. Access barriers to obstetric care at health facilities in sub-Saharan Africa—a systematic review. *Syst Rev*. 2017;6. doi:10.1186/s13643-017-0503-x

16. Sather M, Fajon A-V, Zaentz R, Rubens CE. Global report on preterm birth and stillbirth (5 of 7): advocacy barriers and opportunities. *BMC Pregnancy Childbirth*. 2010;10(1):S5.

17. Sialubanje C, Massar K, Hamer DH, Ruiter RA. Reasons for home delivery and use of traditional birth attendants in rural Zambia: a qualitative study. *BMC Pregnancy Childbirth*. 2015;15(1):216. doi:10.1186/s12884-015-0652-7

18. Mannava P, Durrant K, Fisher J, Chersich M, Luchters S. Attitudes and behaviours of maternal health care providers in interactions with clients: a systematic review. *Glob Health*. 2015;11(1):36. doi:10.1186/s12992-015-0117-9

19. Yakong VN, Rush KL, Bassett-Smith J, Bottorff JL, Robinson C. Women's experiences of seeking reproductive health care in rural Ghana: challenges for maternal health service utilization. *J Adv Nurs*. 2010;66(11):2431-2441. doi:https://doi.org/10.1111/j.1365-2648.2010.05404.x

20. Cheptum JJ, Gitonga MM, Mutua EM, Mukui SJ, Ndambuki JM, Koima WJ. Perception about traditional birth attendants by men and women of reproductive age in rural Migori County, Kenya. *Int J Afr Nurs Sci*. 2017;7:55-61. doi:10.1016/j.ijans.2017.07.002

21. Dayyabu AL, Murtala Y, Grünebaum A, et al. Midwife-assisted planned home birth: an essential component of improving the safety of childbirth in Sub-Saharan Africa. *J Perinat Med*. 2018;47(1):16-21. doi:10.1515/jpm-2018-0066

22. Smith J, Banay R, Zimmerman E, Caetano V, Musheke M, Kamanga A. Barriers to provision of respectful maternity care in Zambia: results from a qualitative study through the lens of behavioral science. *BMC Pregnancy Childbirth*. 2020;20(1):26. doi:10.1186/s12884-019-2579-x

For peer review only - http://bmjopen.bmj.com/site/about/guidelines.xhtml

23. Biemba G, Yeboah-Antwi K, Semrau K, Hammond E, Hamer D. Who is assisting women to deliver babies within health facilities. *Anal Deliv Four Prov Zamb Austin J Public Health Epidemiol*. 2014;1(2):1007.

24. Some TD, Sombie I, Meda N. Women's perceptions of homebirths in two rural medical districts in Burkina Faso: a qualitative study. *Reprod Health*. 2011;8(1):3. doi:10.1186/1742-4755-8-3

25. Berhe R, Nigusie A. Perceptions of Home Delivery Risk and Associated Factors among Pregnant Mothers in North Achefer District, Amhara Region of Ethiopia: The Health Belief Model Perspective. 2019;(238):10.

26. Ajah LO, Onu FA, Ogbuinya OC, et al. Choice of birth place among antenatal clinic attendees in rural mission hospitals in Ebonyi State, South-East Nigeria. *PloS One*. 2019;14(11):e0211306. doi:10.1371/journal.pone.0211306

27. Lwelamira J, Safari J. Choice of Place for Childbirth: Prevalence and Determinants of Health Facility Delivery Among Women in Bahi District, Central Tanzania. *AJMS*. 2011;4.

28. Pardeshi GS, Dalvi SS, Pergulwar CR, Gite RN, Wanje SD. Trends in choosing place of delivery and assistance during delivery in Nanded district, Maharashtra, India. *J Health Popul Nutr*. 2011;29(1):71-76. doi:10.3329/jhpn.v29i1.7568

29. Scott NA, Henry EG, Kaiser JL, et al. Factors affecting home delivery among women living in remote areas of rural Zambia: a cross-sectional, mixed-methods analysis. *Int J Womens Health*. 2018;10:589-601. doi:10.2147/IJWH.S169067

30. Henry EG, Semrau K, Hamer DH, et al. The influence of quality maternity waiting homes on utilization of facilities for delivery in rural Zambia. *Reprod Health*. 2017;14(1):68. doi:10.1186/s12978-017-0328-z

31. Lori JR, Perosky J, Munro-Kramer ML, et al. Maternity waiting homes as part of a comprehensive approach to maternal and newborn care: a cross-sectional survey. *BMC Pregnancy Childbirth*. 2019;19(1):228. doi:10.1186/s12884-019-2384-6

32. Tiruneh GT, Karim AM, Avan BI, et al. The effect of implementation strength of basic emergency obstetric and newborn care (BEmONC) on facility deliveries and the met need for BEmONC at the primary health care level in Ethiopia. *BMC Pregnancy Childbirth*. 2018;18. doi:10.1186/s12884-018-1751-z

33. Bintabara D, Ernest A, Mpondo B. Health facility service availability and readiness to provide basic emergency obstetric and newborn care in a low-resource setting: evidence from a Tanzania National Survey. *BMJ Open*. 2019;9(2):e020608. doi:10.1136/bmjopen-2017-020608

For peer review only - http://bmjopen.bmj.com/site/about/guidelines.xhtml

STROBE Statement—Checklist of items that should be included in reports of ***cohort studies***

	Item No	Recommendation	Page No
**Title and abstract**	1	(*a*) Indicate the study's design with a commonly used term in the title or the abstract	1
		(*b*) Provide in the abstract an informative and balanced summary of what was done and what was found	1
**Introduction**			
Background/rationale	2	Explain the scientific background and rationale for the investigation being reported	1
Objectives	3	State specific objectives, including any prespecified hypotheses	1
**Methods**			
Study design	4	Present key elements of study design early in the paper	1-2
Setting	5	Describe the setting, locations, and relevant dates, including periods of recruitment, exposure, follow-up, and data collection	2-3
Participants	6	(*a*) Give the eligibility criteria, and the sources and methods of selection of participants. Describe methods of follow-up	3
		(*b*) For matched studies, give matching criteria and number of exposed and unexposed	
Variables	7	Clearly define all outcomes, exposures, predictors, potential confounders, and effect modifiers. Give diagnostic criteria, if applicable	4
Data sources/ measurement	8*	For each variable of interest, give sources of data and details of methods of assessment (measurement). Describe comparability of assessment methods if there is more than one group	4
Bias	9	Describe any efforts to address potential sources of bias	3-4
Study size	10	Explain how the study size was arrived at	3-4
Quantitative variables	11	Explain how quantitative variables were handled in the analyses. If applicable, describe which groupings were chosen and why	4-5
Statistical methods	12	(*a*) Describe all statistical methods, including those used to control for confounding	4-5
		(*b*) Describe any methods used to examine subgroups and interactions	
		(*c*) Explain how missing data were addressed	
		(*d*) If applicable, explain how loss to follow-up was addressed	
		(*e*) Describe any sensitivity analyses	
**Results**			
Participants	13*	(a) Report numbers of individuals at each stage of study—eg numbers potentially eligible, examined for eligibility, confirmed eligible, included in the study, completing follow-up, and analysed	5
		(b) Give reasons for non-participation at each stage	
		(c) Consider use of a flow diagram	
Descriptive data	14*	(a) Give characteristics of study participants (eg demographic, clinical, social) and information on exposures and potential confounders	5-13
		(b) Indicate number of participants with missing data for each variable of interest	
		(c) Summarise follow-up time (eg, average and total amount)	
Outcome data	15*	Report numbers of outcome events or summary measures over time	5

For peer review only - http://bmjopen.bmj.com/site/about/guidelines.xhtml

Main results	16	(*a*) Give unadjusted estimates and, if applicable, confounder-adjusted estimates and their precision (eg, 95% confidence interval). Make clear which confounders were adjusted for and why they were included<br>(*b*) Report category boundaries when continuous variables were categorized<br>(*c*) If relevant, consider translating estimates of relative risk into absolute risk for a meaningful time period	7-8
Other analyses	17	Report other analyses done—eg analyses of subgroups and interactions, and sensitivity analyses	n/a
**Discussion**			
Key results	18	Summarise key results with reference to study objectives	13
Limitations	19	Discuss limitations of the study, taking into account sources of potential bias or imprecision. Discuss both direction and magnitude of any potential bias	16
Interpretation	20	Give a cautious overall interpretation of results considering objectives, limitations, multiplicity of analyses, results from similar studies, and other relevant evidence	16
Generalisability	21	Discuss the generalisability (external validity) of the study results	
**Other information**			
Funding	22	Give the source of funding and the role of the funders for the present study and, if applicable, for the original study on which the present article is based	

*Give information separately for exposed and unexposed groups.

Note: An Explanation and Elaboration article discusses each checklist item and gives methodological background and published examples of transparent reporting. The STROBE checklist is best used in conjunction with this article (freely available on the Web sites of PLoS Medicine at http://www.plosmedicine.org/, Annals of Internal Medicine at http://www.annals.org/, and Epidemiology at http://www.epidem.com/). Information on the STROBE Initiative is available at http://www.strobe-statement.org.

For peer review only

BMJ Open

Intended versus actual delivery location and factors associated with change in delivery location among pregnant women in Southern Province, Zambia: a pre-specified secondary observational analysis of the ZamCAT trial

Journal:	*BMJ Open*
Manuscript ID	bmjopen-2021-055288.R1
Article Type:	Original research
Date Submitted by the Author:	21-Dec-2021
Complete List of Authors:	Solomon, Hiwote; Boston University School of Public Health, Doctor of Public Health Program<br>Henry, Elizabeth; Harvard University T H Chan School of Public Health, Department of Global Health and Population; Boston University School of Public Health, Department of Global Health<br>Herlihy, Julie; Boston University School of Medicine, Pediatrics<br>Yeboah-Antwi, Kojo; Fr. Thomas Alan Rooney Memorial Hospital<br>Biemba, Godfrey; Boston University School of Public Health; Zambia National Health Research Authority<br>Musokotwane, Kebby; Levy Mwanawasa Medical University<br>Bhutta, Afsah ; Dow University of Health Sciences<br>Hamer, D.; Boston University School of Public Health, Department of Global Health; Boston University School of Medicine, Department of Medicine<br>Semrau, Katherine; Brigham and Women's Hospital, Ariadne Labs; Brigham and Women's Hospital, Division of Global Health Equity & Department of Medicine
**Primary Subject Heading**:	Public health
Secondary Subject Heading:	Global health, Public health
Keywords:	PUBLIC HEALTH, Maternal medicine < OBSTETRICS, EPIDEMIOLOGY

SCHOLARONE™
Manuscripts

BMJ

I, the Submitting Author has the right to grant and does grant on behalf of all authors of the Work (as defined in the below author licence), an exclusive licence and/or a non-exclusive licence for contributions from authors who are: i) UK Crown employees; ii) where BMJ has agreed a CC-BY licence shall apply, and/or iii) in accordance with the terms applicable for US Federal Government officers or employees acting as part of their official duties; on a worldwide, perpetual, irrevocable, royalty-free basis to BMJ Publishing Group Ltd ("BMJ") its licensees and where the relevant Journal is co-owned by BMJ to the co-owners of the Journal, to publish the Work in this journal and any other BMJ products and to exploit all rights, as set out in our licence.

The Submitting Author accepts and understands that any supply made under these terms is made by BMJ to the Submitting Author unless you are acting as an employee on behalf of your employer or a postgraduate student of an affiliated institution which is paying any applicable article publishing charge ("APC") for Open Access articles. Where the Submitting Author wishes to make the Work available on an Open Access basis (and intends to pay the relevant APC), the terms of reuse of such Open Access shall be governed by a Creative Commons licence – details of these licences and which Creative Commons licence will apply to this Work are set out in our licence referred to above.

Other than as permitted in any relevant BMJ Author's Self Archiving Policies, I confirm this Work has not been accepted for publication elsewhere, is not being considered for publication elsewhere and does not duplicate material already published. I confirm all authors consent to publication of this Work and authorise the granting of this licence.

For peer review only

Intended versus actual delivery location and factors associated with change in delivery location among pregnant women in Southern Province, Zambia: a pre-specified secondary observational analysis of the ZamCAT trial

Hiwote Solomon[1], Elizabeth G Henry[2,3], Julie M Herlihy[4], Kojo Yeboah-Antwi[5], Godfrey Biemba[1,6], Kebby Musokotwane[7], Afsah Bhutta[8], Davidson H. Hamer[3,9], Katherine E.A. Semrau[10,11,12]

[1] Boston University School of Public Health, Boston, Massachusetts, USA
[2] Department of Global Health and Population, Harvard TH Chan School of Public Health, Boston, Massachusetts, USA
[3] Department of Global Health, Boston University School of Public Health, Boston, Massachusetts, USA
[4] Department of Pediatrics, Boston University School of Medicine, Boston, Massachusetts, USA
[5] Fr. Thomas Alan Rooney Memorial Hospital, Asankrangwa, Western Region, Ghana
[6] Zambia National Health Research Authority, Lusaka, Zambia
[7] Levy Mwanawasa University Teaching Hospital, Lusaka, Zambia
[8] Dow University of Health Sciences, Karachi, Pakistan1 Section of Infectious Diseases,
[9] Department of Medicine, Boston University School of Medicine, Boston, MA, USA
[10] Ariadne Labs, Brigham and Women’s Hospital and Harvard TH Chan School of Public Health, Boston, Massachusetts, USA
[11] Division of Global Health Equity & Department of Medicine, Brigham and Women's Hospital, Boston, Massachusetts, USA
[12] Department of Medicine, Harvard Medical School, Boston, Massachusetts, USA

Corresponding author
Hiwote Solomon, MPH
Boston University School of Public Health
801 Massachusetts Avenue
Boston, MA 02119
solomohi@bu.edu

For peer review only

Abstract

Objectives: This pre-specified, secondary analysis of the Zambia Chlorhexidine Application Trial (ZamCAT) aimed to determine the proportion of women who did not deliver where they intended, to understand the underlying reasons for the discordance between planned and actual delivery locations; and to assess sociodemographic characteristics associated with concordance of intention and practice.

Design: Pre-specified, secondary analysis from randomized controlled trial

Setting: Recruitment occurred in 90 primary health facilities (HFs) with follow up in the community in Southern Province, Zambia

Participants: Between Feb 15, 2011, and Jan 30, 2013, 39,679 pregnant women enrolled in ZamCAT.

Secondary outcome measures: The location where mothers gave birth (home vs. HF) was compared to their planned delivery location.

Results: When interviewed antepartum, 92% of respondents intended to deliver at a HF, 6.1% at home, and 1.2% had no plan. However, of those who intended to deliver at a HF, 61% did; of those who intended to deliver at home, only 4% did; and of those who intended to deliver at home, 2% delivered instead at a HF. Among women who delivered at home, women who were aged 25-34 and $\geq$ 35 years were more likely to deliver where they intended than women aged 20-24 years (aOR=1.31, 95% CI: 1.14, 1.50,) and (aOR=1.32, 95% CI: 1.12, 1.57), respectively. Women who delivered at HFs had greater odds of delivering where they intended if they received any primary schooling (aOR=1.34, 95% CI=1.09, 1.72) or more than a primary school education (aOR=1.54, 95% CI=1.17, 2.02), were literate (aOR=1.30, 95% CI=1.09, 1.54), and were not in the lowest quintile of the wealth index.

Conclusion: Discrepancies between intended and actual delivery locations highlight the need to go beyond the development of birth plans and exposure to birth planning messaging. More is required to address barriers to achieving intentions of a facility-based childbirth.

Trial Registration: ClinicalTrials.gov Identifier: NCT01241318

Funding Statement: This secondary analysis research received no specific grant from any funding agency in the public, commercial or not-for-profit sectors. The original data collection and primary trial was funded by the Bill & Melinda Gates Foundation (global health grant number OPPGH5298). The findings and conclusions contained within are those of the authors and do not necessarily reflect positions or policies of the Bill & Melinda Gates Foundation.

Word Count: Abstract: 300; Manuscript: 3,520

For peer review only

For peer review only - http://bmjopen.bmj.com/site/about/guidelines.xhtml

Article Summary

Strengths and limitations of this study:

- In this large, cluster-randomized controlled trial, we were able to gain information on >39,000 pregnant women enrolled with less than 5% loss to follow-up, providing robust estimates of factors related to birth location intentions and actual practice.
- The study was further strengthened by variation in intended location of birth as well as differences in actual practice of delivery location.
- Social desirability bias among respondents as questions such as birth plan and reasons behind delivery choice may have influenced women to respond that they intended to deliver in a facility.
- Qualitative data would be helpful to explore the underlying motivations and decision-making processes around intention and actual delivery locations.

For peer review only

Introduction

Each year, 140 million newborns are born worldwide.[1] While there has been global progress in reducing child mortality, corresponding rates of reduction in newborn mortality have been far slower and annually 2.5 million newborn deaths occur.[2] As the world strives to meet the Sustainable Development Goals (SDGs) by 2030, it is important to understand not only the direct causes, but also health systems barriers and social determinants of health that lead to poor maternal and newborn outcomes.[3] Factors responsible for these outcomes include poverty, illiteracy, issues of women's empowerment and systematic marginalization of communities on the basis of gender, ethnicity or geography.[2,4] In sub-Saharan Africa, birth planning and facility-based childbirth have been core components of interventions to improve maternal and neonatal outcomes. However, this has been insufficient as health systems need to be structurally redesigned to respond to local contexts.[5] Given the emphasis on birth planning in developing countries, it is important to understand why women deliver in locations that differ from what they intended, and what contributes to that change in plan, in order to adequately design interventions and messaging to address specific barriers that prevent women from delivering where they intended.[6–8]

Health facility delivery at primary health facilities with support from referral-level facilities for complicated births has been shown to improve maternal and neonatal outcomes.[5,7,9–11] In order to better understand some of the underlying factors and determinants of access to health facilities equipped with emergency obstetrical and newborn care (EmONC) and skilled birth attendants in rural Zambia, we used data from the Zambia Chlorhexidine Application Trial (ZamCAT), a cluster randomized controlled trial that assessed whether daily 4% chlorohexidine cord cleansing was more effective than dry cord care for prevention of neonatal deaths and omphalitis (umbilical cord infection) in Southern Province.[12] The aim of this pre-specified, secondary analysis was to

For peer review only
For peer review only - http://bmjopen.bmj.com/site/about/guidelines.xhtml

determine the proportion of women who did not deliver where they had intended, to understand the underlying reasons for the discordance between planned and actual delivery locations; and to determine sociodemographic characteristics associated with where pregnant women intended to deliver [i.e. concordance with intention and practice].

Methods

Study Site:

Southern Province, one of ten provinces in Zambia, consists of 13 districts with an estimated population of 2.1 million in 2020.[13] At the time of the ZamCAT study, the 2013-14 Zambia Demographic Health Survey (ZDHS) reported that nearly all women (96%) received at least one antenatal care visit during pregnancy.[14] Additionally, more than half of women (55.9%) in Southern Province delivered in a health facility (predominately public sector facilities), while 41% delivered at home.[14] Skilled birth attendants were present for 55% of all deliveries in Southern Province, while untrained family or relatives assisted with 22% of home deliveries.[14]

Study Design

ZamCAT, conducted from 2011-2013, was a cluster-randomized trial that enrolled and followed a cohort of 39,679 pregnant women living in six districts of Southern Province from before delivery to 28 days after delivery (ClinicalTrials.gov Identifier: NCT01241318). The analysis presented here was an *a priori* research question focusing on one of the ZamCAT study's secondary outcomes, namely: where did pregnant women enrolled in ZamCAT plan to deliver, what were their reasons for their intended location, and what was their actual practice? Our analysis also assessed factors that may have influenced intended and actual delivery locations.

For peer review only - http://bmjopen.bmj.com/site/about/guidelines.xhtml

For peer review only

In the main trial, the units of randomization (clusters) for ZamCAT were Zambian government-run or mission-run primary health-care centers in Southern Province. Ninety clusters were randomized into two groups: (1) clean dry cord care in accordance with Ministry of Health guidelines (control) and (2) daily 4% chlorhexidine cord washes until three days after the umbilical cord fell off (intervention). The study location included a wide range of health facilities, both urban and rural, with differences in staffing and catchment area population characteristics.

Eligible women were in their second or third trimester, aged 15 years or older, planning to stay in the catchment area until 28 days postpartum, and willing to provide informed consent and complete cord care as per cluster assignment.[12] Women were screened by field monitors (data collectors), and women were enrolled either at a facility-based antenatal care visit or through community outreach events. At enrollment, a detailed questionnaire was administered which included questions on household information, birth plan, and antenatal care characteristics. After the enrollment survey was completed, participants were encouraged to deliver at the nearest health facility and were given standard newborn care messages which included information about delivery location, breastfeeding, cord care and danger signs of ill health in their newborn baby as per national and Ministry of Health guidelines. At a home visit two weeks after enrollment, all participants, irrespective of study allocation, were provided a standard clean delivery kit. A birth notification system was developed in collaboration with the community specific to each cluster to alert study team of births.[15] For both study arms, field monitors conducted routine home visits at least once during the antenatal period and four times after delivery (days 1, 4, 10, and 28 postpartum). Detailed information on where the woman delivered and why was collected during the day 4 postpartum visit. Data were collected using paper forms designed in the Teleforms

For peer review only - http://bmjopen.bmj.com/site/about/guidelines.xhtml

system (HP Cambridge, UK). Supervisors reviewed forms for illegible and missing entries and worked with the field monitors to make sure they were complete. The forms were transported to the central study office in Choma where a data management team scanned and entered them into a Microsoft Access Database.

Statistical analysis

Using the full cohort of women enrolled in the study, we assessed women's socioeconomic and pregnancy-related characteristics, and then related them to the outcome of interest (delivery where intended). Our primary outcome was a four-level categorical variable: intended to deliver at health facility and did, intended to deliver at home and did, intended to deliver at a health facility but delivered at home, and intended to deliver at home but delivered at a health facility. Socioeconomic and pregnancy-related characteristics were documented during the enrollment visit and compared across the intention/practice groups. Pregnancy-related characteristics potentially associated with the outcomes were compared between the two groups using the $\chi 2$ test, Fisher's exact test, t-tests, or non-parametric Wilcoxon rank sum tests, as appropriate for continuous or categorical variables. We also assessed the reasons women indicated for why they intended to deliver at a certain location and reasons given after actual delivery on where their actual birth location; women could choose among eight different reasons for why they chose their intended or actual delivery location. Women were allowed to choose more than one reason for why they chose their intended or actual delivery location. All statistical analyses were completed using SAS University Edition®.[16] We assessed the association of sociodemographic characteristics with women delivering where they intended to deliver or elsewhere using logistic regression modeling. We also conducted a stratified

For peer review only - http://bmjopen.bmj.com/site/about/guidelines.xhtml

logistic regression model to assess the predictors of women delivering where they intended stratified by actual delivery location (home and facility).

The Boston University Medical Campus Institutional Review Board (FWA# 00008404) and University of Zambia Research Ethics Committee (FWA# 00001131) provided ethical clearance. The Zambia National Health Research Authority approved the study. All women provided written informed consent, which was obtained in English or Tonga.

Patient and Public Involvement

Study preparations for the overall ZamCAT trial required baseline formative ethnographic research, substantial community sensitization, and engagement with three levels of stakeholders, each necessitating different strategies. The process of community engagement for ZamCAT is detailed further by Hamer et al.[15] Community leaders and neighborhood health committees in the 90 clusters identified appropriate Field Monitor (data collector) candidates according to specific selection criteria. The local health facility staff assisted by ranking lists of potential data collectors provided by the community. Further, the effective birth notification system was developed by the community leaders. Results from the primary study were shared through community meetings in collaboration with the district health offices and at the Zambian National Health Research Conference.

Results

A total of 39,679 pregnant women were enrolled in ZamCAT between Feb 15, 2011 and Jan 30, 2013.[12] During the study 38,131 women had 38,579 deliveries; 1,548 enrolled women were excluded as a result of false pregnancies, miscarriages or abortion, withdrawal from study before

For peer review only - http://bmjopen.bmj.com/site/about/guidelines.xhtml

delivery, lost to follow-up, or death. We had intended and actual delivery information on 37,105 of the enrolled population. The mean age (± standard deviation [SD]) of the women was 25.6 ± 6.9 years, 10% had no formal education, and 52% had only a primary education (Table 1). Of the 37,105 with birth plans stated, 467 (1.2%) had no plan, 2,278 (6.1%) planned to deliver at home, 33,953 (91.5%) intended to deliver at a health facility, and 407 (1.1%) stated other. In practice, 13,139 (35.4%) actually delivered at home, 23,666 (63.7%) actually delivered at a health facility, and 300 (0.8%) delivered in another location.

Table 1. Demographic, household, and pregnancy characteristics of women enrolled in ZamCAT

**Characteristics**	Intended to deliver at health facility and did **n=22 651**	Intended to deliver at home and did **n=1515**	Intended to deliver at health facility but delivered at home **n=11 016**	Intended to deliver at home but delivered at a health facility **n=755**
Maternal Age (years), n (%)				
<20	5919 (26.1%)	185 (12.2%)	2115(19.2%)	215 (28.5%)
20-35	14473 (63.9%)	1114 (73.5%)	7626 (69.2%)	442 (58.5%)
>35	2259 (10.0%)	216 (14.3%)	1275 (11.6%)	98 (13.0%)
Missing				
Tribe, n (%)				
Tonga	19 295 (85.2%)	1431 (94.5%)	10 257 (93.1%)	670 (88.7%)
Ila	142 (0.6%)	7 (0.5%)	53 (0.5%)	4 (0.5%)
Lozi	1175 (5.2%)	20 (1.3%)	238 (2.2%)	28 (3.7%)
Nyanja	687 (3.0%)	18 (1.2%)	147 (1.3%)	13 (1.7%)
Bemba	568 (2.5%)	23 (1.5%)	135 (1.2%)	24 (3.2%)
Other	781 (3.4%)	15 (1.0%)	183 (1.7%)	12 (1.6%)
Missing	3 (0.08%)	1 (0.1%)	3 (0.03%)	4 (0.5%)
Women's Education Level, n (%)				
No education	1681 (7.4%)	265 (17.5%)	1442 (13.1%)	95 (12.6%)
Some primary education	10 779 (47.6%)	1001 (66.1%)	6487 (58.9%)	366 (48.5%)
Some secondary education	9884 (43.6%)	246 (16.2%)	3067 (27.8%)	290 (38.4%)
Higher than secondary education	288 (1.3%)	2 (0.13%)	14 (0.1%)	0 (0.0%)
Unknown or Missing	19 (0.1%)	1 (0.1%)	6 (0.06%)	4 (0.5%)
Maternal Literacy, n (%)				
Illiterate	4875 (21.5%)	611 (40.3%)	3720 (33.8%)	226 (29.9%)
Can read a bit	10620 (46.9%)	716 (47.3%)	5212 (47.4%)	332 (44.0%)
Can read very well	7078 (31.2%)	177 (11.7%)	2022 (18.4%)	191 (25.3%)
No response or Missing	78 (0.3%)	11 (0.7%)	62 (0.6%)	6 (0.8%)
Wealth Index, n (%)				
1-lowest	5214 (23.0%)	541 (35.7%)	3154 (28.6%)	246 (32.6%)
2	5407 (23.9%)	421 (27.8%)	2910 (26.4%)	175 (23.2%)
3	5490 (24.2%)	361 (23.8%)	2807 (25.5%)	181 (24.0%)
4-highest	6540 (28.9%)	192 (12.7%)	2145 (19.5%)	151 (20.0%)

For peer review only - http://bmjopen.bmj.com/site/about/guidelines.xhtml

Marital Status, n (%)				
Single	4173 (18.4%)	82 (5.4%)	1243 (11.3%)	132 (17.5%)
Married or Cohabitating	18 267 (80.7%)	1405 (92.7%)	9644 (87.6%)	600 (79.5%)
Separated, Divorced, or Widowed	204 (0.9%)	26 (1.7%)	128 (1.2%)	19 (2.5%)
Missing	7 (0.03%)	2 (0.1%)	1 (0.01%)	4 (0.5%)
Median household size (IQR)	6 (4-8)	6 (4-8)	6 (4-8)	6 (4-8)
Time walking from home to health facility				
<1h	9276 (41.0%)	293 (19.3%)	2301 (20.9%)	253 (33.5%)
1 to <2 h	7823 (34.5%)	526 (34.7%)	4202 (38.1%)	278 (36.8%)
2 to <3h	3981 (17.6%)	449 (29.6%)	3054 (27.7%)	140 (18.5%)
3 to <4 h	1047 (4.6%)	167 (11.0%)	1017 (9.2%)	58 (7.7%)
4 to <5 h	250 (1.1%)	43 (2.8%)	248 (2.3%)	12 (1.6%)
5 h or more	120 (0.5%)	12 (0.8%)	129 (1.2%)	8 (1.1%)
Unknown	81 (0.4%)	17 (1.1%)	42 (0.4%)	2 (0.3%)
Missing	74 (0.3%)	8 (0.5%)	23 (0.2%)	4 (0.5%)
**Pregnancy Characteristics**				
Parity, mean (SD)	2.7 (2.3)	2.4 (2.3)	2.3 (2.3)	2.5 (2.3)
Primiparous, n (%)	4230 (18.7%)	231 (15.3%)	2188 (19.9%)	119 (15.8%)
Multiparous, n (%)	11 939 (52.7%)	1131 (74.7%)	7086 (64.3%)	401 (53.1%)
Missing, n (%)	6482 (28.6%)	153 (10.1%)	1742 (15.8%)	235 (31.1%)
Gravida, mean (SD)	3.8 (2.4)	3.5 (2.3)	3.4 (2.3)	3.5 (2.3)
Primigravida, n (%)	6327 (27.9%)	148 (9.8%)	1702 (15.5%)	225 (29.8%)
Multigravida, n (%)	16 294 (71.4%)	1362 (89.9%)	9298 (84.4%)	525 (69.5%)
Missing, n (%)	30 (0.1%)	5 (0.3%)	16 (0.2%)	5 (0.7%)
Gestational age at enrollment, mean (SD)	27.9 (6.8)	28.0 (7.0)	28.1 (7.1)	28.1 (7.0)
Time between water breaking and delivery, n (%)				
Less than 24 hours	20,259 (91.7%)	1344 (88.7%)	10,123 (91.9%)	418 (55.4)
24-48 hours	781 (3.5%)	25 (1.7%)	168 (1.5%)	35 (4.6%)
More than 48 hours	120 (0.54%)	8 (0.53%)	26 (0.2%)	2 (0.3%)
Don't know	915 (4.1%)	120 (8.0%)	446 (4.1%)	32 (4.2%)
Missing	576 (2.5%)	1 (1.2%)	253 (2.3%)	268 (35.5%)

*Those who responded to having "no plan" or "other" when asked about intended delivery location, and those who responded as "other" when asked about actual delivery location were excluded from demographic analysis (n=1,168).

Reasons for planned vs. actual delivery location

At study enrollment, respondents were asked about their birth plan and where they planned to deliver as well as why (Table 2). Almost all women had a delivery location in their plan (98.8%) and nearly all women (91.6%) reported that they planned to deliver in a health facility. Women who planned to deliver in a health facility stated they were motivated by safety for mother/baby

(83.9%) and need for skilled attendance (81.3%). Among those who intended to deliver at home, distance was the most commonly noted reason (37.9%) and same location as previous deliveries (29.2%).

Table 2. Planned delivery location and rationale

	No Plan (N=467)		Health Facility (N=33,953)		Home (N=2,278)		Other (N=407)	
	n	%	n	%	n	%	n	%
Same location as prior delivery	3	0.6%	4,237	12.5%	666	29.2%	55	13.5%
Need for skilled attendance	14	3.0%	27,578	81.2%	402	17.7%	281	69.0%
Financial restraints	13	2.8%	1,058	3.1%	443	19.5%	16	3.9%
Distance	10	2.1%	4,714	13.9%	851	37.4%	45	11.1%
Relationship with Providers	3	0.8%	453	1.3%	175	7.7%	8	2.0%
Family/social expectations	2	0.5%	714	2.1%	264	11.6%	2	0.5%
Safety for mother/baby	12	2.6%	28,548	84.1%	464	20.4%	293	72.0%
Other	3	0.6%	276	0.8%	47	2.1%	33	8.1%
Missing	0	0.0%	1	0.0%	5	0.2%	4	1.0%

**Note "Other" in birth plan often includes HOSPITAL delivery which is separate from health facility delivery as well as TBA delivery.*

After delivery, women were asked about where they actually delivered and why (Table 3). Although nearly all women indicated that they planned to deliver at a health facility, only 63.8% (N=23,666) actually did. The primary motivations for delivery in the facility was need for skilled attendance (54.6%) and safety for the mother and baby (55.1%). While only 6% of women intended to deliver at home, over one-third (35.4%) of women actually did (N=13,139). The top three reasons for delivering at home were distance (29.7%), need for skilled attendance (43.0%), and safety for mother/baby (44.7%).

For peer review only - http://bmjopen.bmj.com/site/about/guidelines.xhtml

Table 3. Actual delivery location and reasoning

	Health Facility (N=23,666)		Home (N=13,139)		Other (N=300)	
	n	%	n	%	n	%
Same location as prior delivery	3,182	13.5%	2,062	15.7%	47	15.7%
Need for skilled attendance	12,916	54.6%	5,654	43.0%	164	54.7%
Financial restraints	1799	7.6%	2,234	9.4%	13	4.3%
Distance	6,303	26.6%	3,898	29.7%	74	24.7%
Relationship with Providers	571	2.4%	426	3.2%	9	3.0%
Family/social expectations	1,251	5.3%	961	7.5%	17	5.7%
Safety for mother/baby	13,048	55.1%	5,866	44.7%	170	56.7%
Other	1,468	6.2%	792	6.0%	23	7.7%
Missing	1,353	5.7%	762	5.8%	15	5.0%

Note: Health facility in actual delivery location includes hospital delivery

Intended vs. actual delivery location

Overall, 67% of women delivered in their intended location. Almost two-thirds of women (n=22,651, 61.1%) who intended to deliver at a health facility actually did (concordant); in contrast, almost one-third of women who intended to deliver at a health facility (n=11,016, 29.7%) delivered at home (discordant). Of those who intended to deliver at home, only 4.1% (n=1515) actually did. Additionally, of those women who intended to deliver at home, 2.0% (n=755) delivered instead at a health facility. Excluded were those who indicated “no plan” or “other” for intended delivery location and those who indicated “other” for actual delivery location.

We observed that 18.7% of those who intended to deliver at a heath facility and did were primiparous, compared to women who intended to deliver at a health facility but delivered at home (19.9%). For those women who were multiparous, more than half (52.7%) intended to deliver at home and actually did; two-thirds (64.3%) of women who intended to deliver at a health facility, delivered at home. We found that time between water breaking and delivering in less than 24 hours was not shorter for those who intended to delivery at a health facility but delivered at home.

For peer review only - http://bmjopen.bmj.com/site/about/guidelines.xhtml

Women who intended to deliver at home and subsequently delivered at a health center stated they needed a skilled attendant for the safety of the mother and baby (Table 4). About 30% of respondents, who indicated distance as a reason for delivering where they did, delivered at home. For those who intended to deliver at a health facility and did, 41% lived less than a one hour walk from the health facility. Of women who intended to deliver at home and did, 64% lived between one to three hours walk from the nearest health facility. Among those who intended to deliver at a health facility and actually delivered at home, 46.3% indicated that they did so because of safety for mother/baby. Additionally, this same group indicated a need for skilled attendance as a reason for delivering at home versus at a health facility. Among those who intended to and actually delivered at home, only 8.9% indicated family/social expectations as a reason while 34.5% indicated safety for mother/baby as a reason.

Table 4. Comparison of Intended and Actual Delivery Location, with Reasons for Actual Delivery Location

Reasons for Actual Delivery Location	Intended to deliver at health facility and did (N=22 651)		Intended to deliver at home and did (N=1515)		Intended to deliver at health facility but delivered at home (N=11 016)		Intended to deliver at home but delivered at a health facility (N=755)	
	**n**	**%**	**n**	**%**	**n**	**%**	**n**	**%**
Same location as prior delivery	3020	13.4%	279	18.4%	1677	15.2%	120	15.9%
Need for skilled attendance	12458	54.5%	476	31.4%	4947	44.9%	353	46.8%
Financial restraints	1708	7.7%	149	9.8%	1021	9.3%	72	9.5%
Distance	5987	26.6%	505	33.3%	3229	29.3%	233	30.9%
Relationship with Providers	528	2.4%	76	5.0%	326	3.0%	34	4.5%
Family/social expectations	1182	5.3%	135	8.9%	782	7.1%	48	6.4%
Safety for mother/baby	12585	36.9%	523	34.5%	5095	46.3%	332	44.0%
Other	1408	6.2%	107	7.1%	654	5.9%	44	5.8%
Missing	1296	6.2%	89	5.9%	628	5.7%	47	6.2%
**Time walking from home to health facility**								
<1h	9276	41.0%	293	19.3%	2301	20.9%	253	33.5%
1 to <2 h	7823	34.5%	526	34.7%	4202	38.1%	278	36.8%
2 to <3h	3981	17.6%	449	29.6%	3054	27.7%	140	18.5%
3 to <4 h	1047	4.6%	167	11.0%	1017	9.2%	58	7.7%

For peer review only - http://bmjopen.bmj.com/site/about/guidelines.xhtml

4 to <5 h	250	1.1%	43	2.8%	248	2.3%	12	1.6%
5 h or more	120	0.5%	12	0.8%	129	1.2%	8	1.1%
Unknown	81	0.4%	17	1.1%	42	0.4%	2	0.3%
Missing	74	0.3%	8	0.5%	23	0.2%	4	0.5%

Predictors of women delivering where intended

Table 5 shows the result of our stratified logistic regression model where we assessed predictors of women delivering where they intended to, stratified by home delivery and facility delivery. Among women who delivered at home, we found that women who were aged 25-34 and ≥ 35 years were more likely to deliver where they intended than women aged 20-24 years (aOR=1.31, (95% CI=1.14, 1.50), p=0.17) and (aOR=1.32, (95% CI=1.12, 1.57), p=0.003), respectively. Women who were 15-19 years and delivered at home had lower odds (aOR=0.77, (95% CI=0.60, 0.93), p<.0001) of delivering where they intended compared to those aged 20-24 years. Women who delivered at home and had more than a primary education were less likely to deliver where they intended when compared to those with no education (aOR=0.55, (95% CI=0.43, 0.69), p<0.0001). Those who delivered at home and were not in the lowest wealth quintile were less likely to deliver where they intended (Table 5). When examining the time to walk to nearest health facility, we found that those who delivered at home had higher odds of delivering where they intended to if they lived two to less than three hours from nearest health facility (aOR=1.16, (95% CI=1.02, 1.33), p=0.03) or three to less than four hours away from nearest health facility (aOR=1.27, (95% CI=1.05, 1.54), p=0.01).

For peer review only

Women who delivered at the facility had greater odds of delivering where they intended if they received any primary schooling (aOR=1.34, (95% CI=1.09, 1.72), p=0.008) or more than a primary school education (aOR=1.54, (95% CI=1.17, 2.02), p=0.002), were literate (aOR=1.30 (95% CI=1.09, 1.54), p=0.003), were in the second (aOR=1.37, (95% CI=1.16, 1.63), p=0.0003), third (aOR=1.34, (95% CI: (1.13, 1.58), p=0.0007), or fourth (aOR=1.69, (95% CI=1.40, 2.04), p<0.0001) quintile of the wealth index, and those who experienced headache as a complication during pregnancy (aOR=1.71, (95% CI=1.12, 2.60), p=0.01). In contrast, women who delivered at health facility had lower odds of delivering where they intended if it was their first pregnancy (aOR=0.67, (95% CI=0.55, 0.80), p<0.0001) compared to those who had been pregnant before, lived between a three to less than four hour walk from the nearest health facility (aOR=0.74, (95% CI=0.57, 0.96), p=0.02) compared to those who lived a one to less than two hour walk, lapsed 24-48 hours between water breaking and delivery (aOR=0.534 (95% CI=0.40, 0.73), p<0.0001) compared to those who only lapsed less than 24 hours, and those who experienced bleeding (aOR=0.60, (95% CI=0.40, 0.90), p=0.01), fits (aOR=0.40, (95% CI=0.29, 0.85), p=0.02), vaginal discharge (aOR=0.24, (95% CI=0.13, 0.42), p<.0001), and/or heavy bleeding (aOR=0.73, (95% CI=0.56, 0.97), p=0.03) compared to those who did not experience these symptoms.

For peer review only

For peer review only - http://bmjopen.bmj.com/site/about/guidelines.xhtml

Table 5. Predictors of women delivering where they intended to, stratified by delivery location

Characteristics	Among those that delivered at home, adjusted odds ratio (aOR) of delivering where intended		Among those that delivered at facility, adjusted odds ratio (aOR) of delivering where intended	
	aOR (95% CI)	p-value	aOR (95% CI)	p-value
Maternal Age (years)				
15-19	**0.77 (0.64 0.93)**	**0.007**	**0.69 (0.57, 0.82)**	**<.0001**
20-24	ref		ref	
25-34	**1.31 (1.14, 1.50)**	**<.0001**	0.89 (0.75, 1.05)	**0.17**
>35	**1.32 (1.12, 1.57)**	**0.001**	**0.73 (0.59, 0.90)**	**0.003**
Women's Education Level				
No education	ref		ref	
Any primary	0.90 (0.75, 1.06)	0.21	**1.34 (1.09, 1.72)**	**0.008**
More than primary	**0.55 (0.43, 0.69)**	**<.0001**	**1.54 (1.17, 2.02)**	**0.002**
Marital Status				
Married	ref		ref	
Not married	**0.67 (0.55, 0.83)**	**0.0002**	0.89 (0.76, 1.06)	0.18
Can read				
No	ref		ref	
Yes	1.05 (0.91, 1.20)	0.50	**1.30 (1.09, 1.54)**	**0.003**
Household Light Source				
No electricity	ref		ref	
Electricity	0.81 (0.57, 1.16)	0.25	1.27 (0.95, 1.69)	0.11
Wealth Index				
1-lowest	**ref**	**ref**	**ref**	**ref**
2	**0.85 (0.74, 0.97)**	**0.02**	**1.37 (1.16, 1.63)**	**0.0003**
3	**0.76 (0.66, 0.88)**	**0.0002**	**1.34 (1.13, 1.58)**	**0.0007**
4-highest	**0.56 (0.46, 0.67)**	**<.0001**	**1.69 (1.40, 2.04)**	**<0.0001**
Primigravida				
No	ref		ref	
Yes	0.90 (0.73, 1.11)	0.33	**0.67 (0.55, 0.80)**	**<0.0001**
Time to walk to nearest health facility				
<1h	1.10 (0.94, 1.28)	0.24	1.14 (0.98, 1.32)	0.10
1 to <2 h	ref		ref	ref
2 to <3h	**1.16 (1.02, 1.33)**	**0.03**	0.92 (0.78, 1.09)	0.34
3 to <4 h	**1.27 (1.05, 1.54)**	**0.01**	**0.74 (0.57, 0.96)**	**0.02**
4 to <5 h	1.20 (0.84, 1.68)	0.32	0.80 (0.48, 1.35)	0.41
5 h or more	0.68 (0.37, 1.24)	0.20	0.70 (0.34, 1.45)	0.34
Time between water breaking and delivery				
Less than 24 hours	**ref**		ref	
24-48 hours	1.04 (0.68, 1.59)	0.87	**0.54 (0.40, 0.73)**	**<0.0001**
More than 48 hours	2.11 (0.95, 4.69)	0.07	1.34 (0.44, 4.38)	0.58
Don't know	**1.93 (1.56, 2.38)**	**<.0001**	**0.49 (0.37, 0.64)**	**<0.0001**
Complications during pregnancy*				

High fever	1.45 (1.00, 2.11)	0.05	0.79 (0.54, 1.16)	0.23
Bleeding	1.23 (0.80, 2.05)	0.31	**0.60 (0.40, 0.90)**	**0.01**
Fits	2.15 (0.79, 5.82)	0.13	**0.40 (0.19, 0.85)**	**0.02**
Headache	1.07 (0.79, 1.44)	0.67	**1.71 (1.12, 2.60)**	**0.01**
Vaginal discharge	0.50 (0.15, 1.61)	0.24	**0.24 (0.13, 0.42)**	**<0.0001**
Heavy bleeding	1.08 (0.78, 1.50)	0.63	**0.73 (0.56, 0.97)**	**0.03**
Swelling	0.88 (0.63, 1.22)	0.43	0.82 (0.62, 1.10)	0.19

* Note: Referent groups for complications were the absence of specified complications
**Adjusted for maternal age, women's education, marital status, literacy, wealth index, housing light source

Discussion

Overall, birth plans were widely prepared in this cohort and >90% of women indicated a plan to deliver at a health facility, consistent with the 2013-2014 Zambia Demographic Health Survey (ZDHS). [14] However, only two-thirds of women had actual delivery location in line with their birth plan and >35% of women delivered at home. Our analysis demonstrates clear deviations from the birth plan during the actual delivery, especially among those who had expressed an intent to deliver at a facility. Discrepancy between planning and actual delivery behavior highlights that the development of birth plans and exposure to birth planning messaging are insufficient to address barriers to adhering to plans to have a facility-based childbirth.

Several reasons may underlie the discrepancy between intended location and actual practice around childbirth. One potential reason could be due to women's increased concerns about safety at facilities that are poorer quality. Interestingly, 46% of women in our sample indicated safety for mother/baby as a reason for why they delivered at home even though they intended to deliver at a health facility. Unfortunately, we do not know if this was driven by fear of poor treatment at the facility or fear of transporting/moving the laboring women. In some instances, poorly functioning health systems are unable to engage women in antenatal and post-natal care to create sufficient continuity of care.[17] Some of these barriers may relate to a sense of fatalism around maternal and

For peer review only

newborn care, and are important contributors to preventable mortality.[18] Such disparities present a major challenge to policies and programs aiming to mitigate the burden of maternal and neonatal mortality. The large proportion of facility births in unclean, unhygienic environments, and poor postnatal care may be important contributors to why women indicate safety for mother/baby as a reason why they changed their birth plan from intending to deliver at a health facility to delivering at home.

Another factor that may contribute to discrepancy between intention and practice is the dynamic between provider and patient.[19–21] Among those who intended to deliver at a health facility but instead delivered at home, 45% indicated need for skilled delivery as a reason. While this seems counterintuitive, one reason for this may be the presence of traditional birth attendants (TBAs) in the community who women regard as skilled attendants.[22,23] Although a policy change in Zambia resulted in TBAs no longer being trained and recommended that they stop conducting deliveries at home, a study on reasons for home delivery and use of TBAs in rural Zambia by Sialubanje et al. found that most women had positive attitudes towards TBAs and regarded them as respectful, skilled, and trustworthy.[19] Moreover, most women indicated that negative attitudes toward the quality of services at the clinic as a reason for why they delivered at home.[19] Smith et al. focused on behavioral drivers in labor and delivery wards in Chipata District in Eastern Province, Zambia and found that women in the wards are often subjected to disrespect and abuse.[24] Another study in rural Kenya found that although the community was aware of the risks of delivering with a TBA, many still preferred to deliver with a TBA due to their availability, accessibility, and their friendly attitude.[22] In Zambia, a study by Biemba et al. which utilized data from the ZamCAT study, found that TBAs often end up tending to deliveries even in health facilities due to a lack of qualified

For peer review only - http://bmjopen.bmj.com/site/about/guidelines.xhtml

personnel.[25] As for the safety of mother and child, some women perceive delivering at the health facility as unsafe based on cultural values of elder women in the community.[26] Finally, in another study, women in Ethiopia who had low perceived susceptibility and severity were more likely to intend to deliver at home instead of at a health facility.[26,27]

Among women who intended to deliver at home distance was most often cited as a reason (37.9%), although this less often reported when women were asked why they delivered home post-delivery. Our data are similar to the 2013-14 ZDHS which reported that 32.2% of women in Southern Province indicated distance and lack of transportation as a reason for not delivering in a health facility. Additionally, a study in Ebonyi State, Nigeria on the choice of birth place among antenatal clinic attendees in rural mission hospitals also found that distance served as a major determinant of birth place location.[28] Distance as a barrier to accessing care is not uncommon in many resource-constrained countries in sub-Saharan Africa and Asia.[29,30] Scott el al. found that women who lived in remote areas of Zambia faced persistent challenges regarding delivery location.[31] The study which included four districts in Southern Province also found women who lived far from a health facility, attended fewer antenatal visits, were multigravida, and who did not utilize maternity waiting homes had higher odds of home delivery.[31] Maternal waiting homes have shown to increase likelihood of delivering at a health facility.[31–33] Given that most maternal waiting homes were renovated or built after 2013, our study did not collect data on how maternal waiting home influenced delivery locations for those further away from the health facility.

Our analysis is strengthened by the sample size and variation in adherence to intended delivery location. However, a key limitation is a likelihood for social desirability bias among respondents

For peer review only - http://bmjopen.bmj.com/site/about/guidelines.xhtml

as questions such as birth plan and reasons behind delivery choice may have influenced women to respond they intended to deliver in a facility. Another limitation is that the data are seven years old and may not reflect current trends in delivery in Zambia, which has been affected by COVID-19.[34] Further, we do not have qualitative data could have helped explore the underlying motivations around intention and actual delivery locations.

Understanding factors that influence women to change their birth plan as well as addressing structural and perceived barriers to facility delivery are important to improving care. Maternal and neonatal outcomes can be significantly improved if women delivered at a facility with at least basic emergency obstetric and newborn care.[35,36] Potential interventions that can address barriers to reaching facilities include provision of maternity waiting homes, addressing perceived safety and treatment of mothers and infants in facilities, supporting providers to provide high-quality/respectful maternity care, systemwide plans that support transport of pregnant women to a health facility, messaging around saving for transport to a health facility, further health education for those who are primigravida to deliver a health facility, and safe delivery incentive programs.

Contribution statement:

As the original co-investigators of the ZamCAT trial, DHH and KEAS were responsible for conceiving the original research question, designing the trial, and overseeing the study implementation. KS, DHH and HS were responsible for the conceptualization of this sub-analysis. HS was responsible for the methodology, conducting formal analysis, and the writing and revision of the manuscript. JMH, GB, KM and KY-A were part of the original research team for ZamCAT and were responsible for reviewing and editing the manuscript. EGH was

For peer review only - http://bmjopen.bmj.com/site/about/guidelines.xhtml

responsible for data set creation; preliminary analysis and manuscript review. AB assisted with preliminary analysis, reviewing, and editing of the manuscript.

Competing interests: None declared.

Data Sharing Statement: The data that support the findings of this study are available from the corresponding author, upon reasonable request.

For peer review only

For peer review only - http://bmjopen.bmj.com/site/about/guidelines.xhtml

References

1. Graham W, Woodd S, Byass P, et al. Diversity and divergence: the dynamic burden of poor maternal health. *The Lancet*. 2016;388(10056):2164-2175.

2. UNICEF. Neonatal mortality. UNICEF DATA. Published 2018. Accessed May 13, 2020. https://data.unicef.org/topic/child-survival/neonatal-mortality/

3. United Nations. Transforming our world: the 2030 Agenda for Sustainable Development | Department of Economic and Social Affairs. Published 2015. Accessed January 9, 2021. https://sdgs.un.org/2030agenda

4. Liu L, Oza S, Hogan D, et al. Global, regional, and national causes of under-5 mortality in 2000–15: an updated systematic analysis with implications for the Sustainable Development Goals. *The Lancet*. 2016;388(10063):3027-3035.

5. Hanson C, Waiswa P, Pembe A, Sandall J, Schellenberg J. Health system redesign for equity in maternal and newborn health must be codesigned, country led, adapted to context and fit for purpose. *BMJ Glob Health*. 2020;5(10):e003748. doi:10.1136/bmjgh-2020-003748

6. McCauley H, Lowe K, Furtado N, Mangiaterra V, van den Broek N. What are the essential components of Antenatal Care? A systematic review of the literature and development of signal functions to guide monitoring and evaluation. *BJOG Int J Obstet Gynaecol*. Published online November 28, 2021. doi:10.1111/1471-0528.17029

7. Campbell OMR, Graham WJ, Lancet Maternal Survival Series steering group. Strategies for reducing maternal mortality: getting on with what works. *Lancet Lond Engl*. 2006;368(9543):1284-1299. doi:10.1016/S0140-6736(06)69381-1

8. Bhutta ZA, Das JK, Bahl R, et al. Can available interventions end preventable deaths in mothers, newborn babies, and stillbirths, and at what cost? *Lancet Lond Engl*. 2014;384(9940):347-370. doi:10.1016/S0140-6736(14)60792-3

9. Doctor HV, Nkhana-Salimu S, Abdulsalam-Anibilowo M. Health facility delivery in sub-Saharan Africa: successes, challenges, and implications for the 2030 development agenda. *BMC Public Health*. 2018;18(1):765.

10. Pervin J, Moran A, Rahman M, et al. Association of antenatal care with facility delivery and perinatal survival–a population-based study in Bangladesh. *BMC Pregnancy Childbirth*. 2012;12(1):111.

11. Roder-DeWan S, Nimako K, Twum-Danso NAY, Amatya A, Langer A, Kruk M. Health system redesign for maternal and newborn survival: rethinking care models to close the global equity gap. *BMJ Glob Health*. 2020;5(10):e002539. doi:10.1136/bmjgh-2020-002539

For peer review only - http://bmjopen.bmj.com/site/about/guidelines.xhtml

12. Semrau KEA, Herlihy J, Grogan C, et al. Effectiveness of 4% chlorhexidine umbilical cord care on neonatal mortality in Southern Province, Zambia (ZamCAT): a cluster-randomised controlled trial. *Lancet Glob Health*. 2016;4(11):e827-e836. doi:10.1016/S2214-109X(16)30215-7

13. Zambia Statistics Agency. Population and Demographic Projections, 2011-2035 - Zambia Data Portal. Published 2020. Accessed January 31, 2021. https://zambia.opendataforafrica.org/ZMPHC2015/population-and-demographic-projections-2011-2035

14. Central Statistical Office/Zambia, Ministry of Health/Zambia, University of Zambia Teaching Hospital Virology Laboratory, University of Zambia Department of Population Studies, Tropical Diseases Research Centre/Zambia, I. C. F. International. Zambia Demographic and Health Survey 2013-14. Published online March 1, 2015. Accessed February 14, 2020. https://dhsprogram.com/publications/publication-fr304-dhs-final-reports.cfm

15. Hamer DH, Herlihy JM, Musokotwane K, et al. Engagement of the community, traditional leaders, and public health system in the design and implementation of a large community-based, cluster-randomized trial of umbilical cord care in Zambia. *Am J Trop Med Hyg*. 2015;92(3):666-672.

16. SAS Institute. *SAS University Edition*.; 2020. Accessed January 9, 2021. https://support.sas.com/en/software/university-edition-support.html

17. Kyei-Nimakoh M, Carolan-Olah M, McCann TV. Access barriers to obstetric care at health facilities in sub-Saharan Africa—a systematic review. *Syst Rev*. 2017;6. doi:10.1186/s13643-017-0503-x

18. Sather M, Fajon AV, Zaentz R, Rubens CE. Global report on preterm birth and stillbirth (5 of 7): advocacy barriers and opportunities. *BMC Pregnancy Childbirth*. 2010;10(1):S5.

19. Sialubanje C, Massar K, Hamer DH, Ruiter RA. Reasons for home delivery and use of traditional birth attendants in rural Zambia: a qualitative study. *BMC Pregnancy Childbirth*. 2015;15(1):216. doi:10.1186/s12884-015-0652-7

20. Mannava P, Durrant K, Fisher J, Chersich M, Luchters S. Attitudes and behaviours of maternal health care providers in interactions with clients: a systematic review. *Glob Health*. 2015;11(1):36. doi:10.1186/s12992-015-0117-9

21. Yakong VN, Rush KL, Bassett-Smith J, Bottorff JL, Robinson C. Women's experiences of seeking reproductive health care in rural Ghana: challenges for maternal health service utilization. *J Adv Nurs*. 2010;66(11):2431-2441. doi:https://doi.org/10.1111/j.1365-2648.2010.05404.x

22. Cheptum JJ, Gitonga MM, Mutua EM, Mukui SJ, Ndambuki JM, Koima WJ. Perception about traditional birth attendants by men and women of reproductive age in rural Migori County, Kenya. *Int J Afr Nurs Sci*. 2017;7:55-61. doi:10.1016/j.ijans.2017.07.002

For peer review only - http://bmjopen.bmj.com/site/about/guidelines.xhtml

23. Dayyabu AL, Murtala Y, Grünebaum A, et al. Midwife-assisted planned home birth: an essential component of improving the safety of childbirth in Sub-Saharan Africa. *J Perinat Med*. 2018;47(1):16-21. doi:10.1515/jpm-2018-0066

24. Smith J, Banay R, Zimmerman E, Caetano V, Musheke M, Kamanga A. Barriers to provision of respectful maternity care in Zambia: results from a qualitative study through the lens of behavioral science. *BMC Pregnancy Childbirth*. 2020;20(1):26. doi:10.1186/s12884-019-2579-x

25. Biemba G, Yeboah-Antwi K, Semrau K, Hammond E, Hamer D. Who is assisting women to deliver babies within health facilities. *Anal Deliv Four Prov Zamb Austin J Public Health Epidemiol*. 2014;1(2):1007.

26. Some TD, Sombie I, Meda N. Women's perceptions of homebirths in two rural medical districts in Burkina Faso: a qualitative study. *Reprod Health*. 2011;8(1):3. doi:10.1186/1742-4755-8-3

27. Berhe R, Nigusie A. Perceptions of Home Delivery Risk and Associated Factors among Pregnant Mothers in North Achefer District, Amhara Region of Ethiopia: The Health Belief Model Perspective. 2019;(238):10.

28. Ajah LO, Onu FA, Ogbuinya OC, et al. Choice of birth place among antenatal clinic attendees in rural mission hospitals in Ebonyi State, South-East Nigeria. *PloS One*. 2019;14(11):e0211306. doi:10.1371/journal.pone.0211306

29. Lwelamira J, Safari J. Choice of Place for Childbirth: Prevalence and Determinants of Health Facility Delivery Among Women in Bahi District, Central Tanzania. *AJMS*. 2011;4.

30. Pardeshi GS, Dalvi SS, Pergulwar CR, Gite RN, Wanje SD. Trends in choosing place of delivery and assistance during delivery in Nanded district, Maharashtra, India. *J Health Popul Nutr*. 2011;29(1):71-76. doi:10.3329/jhpn.v29i1.7568

31. Scott NA, Henry EG, Kaiser JL, et al. Factors affecting home delivery among women living in remote areas of rural Zambia: a cross-sectional, mixed-methods analysis. *Int J Womens Health*. 2018;10:589-601. doi:10.2147/IJWH.S169067

32. Henry EG, Semrau K, Hamer DH, et al. The influence of quality maternity waiting homes on utilization of facilities for delivery in rural Zambia. *Reprod Health*. 2017;14(1):68. doi:10.1186/s12978-017-0328-z

33. Lori JR, Perosky J, Munro-Kramer ML, et al. Maternity waiting homes as part of a comprehensive approach to maternal and newborn care: a cross-sectional survey. *BMC Pregnancy Childbirth*. 2019;19(1):228. doi:10.1186/s12884-019-2384-6

34. Amref Health Africa. Impact of the Covid-19 Pandemic and Response on Reproductive, Maternal, Child and Adolescent Health Service Provision in Zambia. Amref Health Africa. Published 2021. Accessed December 13, 2021. https://amref.org/download/impact-of-the-

For peer review only - http://bmjopen.bmj.com/site/about/guidelines.xhtml

covid-19-pandemic-and-response-on-reproductive-maternal-child-and-adolescent-health-service-provision-in-zambia/

35. Tiruneh GT, Karim AM, Avan BI, et al. The effect of implementation strength of basic emergency obstetric and newborn care (BEmONC) on facility deliveries and the met need for BEmONC at the primary health care level in Ethiopia. *BMC Pregnancy Childbirth*. 2018;18. doi:10.1186/s12884-018-1751-z

36. Bintabara D, Ernest A, Mpondo B. Health facility service availability and readiness to provide basic emergency obstetric and newborn care in a low-resource setting: evidence from a Tanzania National Survey. *BMJ Open*. 2019;9(2):e020608. doi:10.1136/bmjopen-2017-020608

For peer review only

For peer review only - http://bmjopen.bmj.com/site/about/guidelines.xhtml

STROBE Statement—Checklist of items that should be included in reports of ***cohort studies***

	Item No	Recommendation	Page No
**Title and abstract**	1	(*a*) Indicate the study's design with a commonly used term in the title or the abstract	1
		(*b*) Provide in the abstract an informative and balanced summary of what was done and what was found	1
**Introduction**			
Background/rationale	2	Explain the scientific background and rationale for the investigation being reported	1
Objectives	3	State specific objectives, including any prespecified hypotheses	1
**Methods**			
Study design	4	Present key elements of study design early in the paper	1-2
Setting	5	Describe the setting, locations, and relevant dates, including periods of recruitment, exposure, follow-up, and data collection	2-3
Participants	6	(*a*) Give the eligibility criteria, and the sources and methods of selection of participants. Describe methods of follow-up	3
		(*b*) For matched studies, give matching criteria and number of exposed and unexposed	
Variables	7	Clearly define all outcomes, exposures, predictors, potential confounders, and effect modifiers. Give diagnostic criteria, if applicable	4
Data sources/ measurement	8*	For each variable of interest, give sources of data and details of methods of assessment (measurement). Describe comparability of assessment methods if there is more than one group	4
Bias	9	Describe any efforts to address potential sources of bias	3-4
Study size	10	Explain how the study size was arrived at	3-4
Quantitative variables	11	Explain how quantitative variables were handled in the analyses. If applicable, describe which groupings were chosen and why	4-5
Statistical methods	12	(*a*) Describe all statistical methods, including those used to control for confounding	4-5
		(*b*) Describe any methods used to examine subgroups and interactions	
		(*c*) Explain how missing data were addressed	
		(*d*) If applicable, explain how loss to follow-up was addressed	
		(*e*) Describe any sensitivity analyses	
**Results**			
Participants	13*	(a) Report numbers of individuals at each stage of study—eg numbers potentially eligible, examined for eligibility, confirmed eligible, included in the study, completing follow-up, and analysed	5
		(b) Give reasons for non-participation at each stage	
		(c) Consider use of a flow diagram	
Descriptive data	14*	(a) Give characteristics of study participants (eg demographic, clinical, social) and information on exposures and potential confounders	5-13
		(b) Indicate number of participants with missing data for each variable of interest	
		(c) Summarise follow-up time (eg, average and total amount)	
Outcome data	15*	Report numbers of outcome events or summary measures over time	5

For peer review only - http://bmjopen.bmj.com/site/about/guidelines.xhtml

Main results	16	(*a*) Give unadjusted estimates and, if applicable, confounder-adjusted estimates and their precision (eg, 95% confidence interval). Make clear which confounders were adjusted for and why they were included<br>(*b*) Report category boundaries when continuous variables were categorized<br>(*c*) If relevant, consider translating estimates of relative risk into absolute risk for a meaningful time period	7-8
Other analyses	17	Report other analyses done—eg analyses of subgroups and interactions, and sensitivity analyses	n/a
**Discussion**			
Key results	18	Summarise key results with reference to study objectives	13
Limitations	19	Discuss limitations of the study, taking into account sources of potential bias or imprecision. Discuss both direction and magnitude of any potential bias	16
Interpretation	20	Give a cautious overall interpretation of results considering objectives, limitations, multiplicity of analyses, results from similar studies, and other relevant evidence	16
Generalisability	21	Discuss the generalisability (external validity) of the study results	
**Other information**			
Funding	22	Give the source of funding and the role of the funders for the present study and, if applicable, for the original study on which the present article is based	

*Give information separately for exposed and unexposed groups.

Note: An Explanation and Elaboration article discusses each checklist item and gives methodological background and published examples of transparent reporting. The STROBE checklist is best used in conjunction with this article (freely available on the Web sites of PLoS Medicine at http://www.plosmedicine.org/, Annals of Internal Medicine at http://www.annals.org/, and Epidemiology at http://www.epidem.com/). Information on the STROBE Initiative is available at http://www.strobe-statement.org.

For peer review only

BMJ Open

Intended versus actual delivery location and factors associated with change in delivery location among pregnant women in Southern Province, Zambia: a pre-specified secondary observational analysis of the ZamCAT trial

Journal:	*BMJ Open*
Manuscript ID	bmjopen-2021-055288.R2
Article Type:	Original research
Date Submitted by the Author:	04-Feb-2022
Complete List of Authors:	Solomon, Hiwote; Boston University School of Public Health, Doctor of Public Health Program<br>Henry, Elizabeth; Harvard University T H Chan School of Public Health, Department of Global Health and Population; Boston University School of Public Health, Department of Global Health<br>Herlihy, Julie; Boston University School of Medicine, Pediatrics<br>Yeboah-Antwi, Kojo; Fr. Thomas Alan Rooney Memorial Hospital<br>Biemba, Godfrey; Boston University School of Public Health; Zambia National Health Research Authority<br>Musokotwane, Kebby; Levy Mwanawasa Medical University<br>Bhutta, Afsah ; Dow University of Health Sciences<br>Hamer, D.; Boston University School of Public Health, Department of Global Health; Boston University School of Medicine, Department of Medicine<br>Semrau, Katherine; Brigham and Women's Hospital, Ariadne Labs; Brigham and Women's Hospital, Division of Global Health Equity & Department of Medicine
**Primary Subject Heading**:	Public health
Secondary Subject Heading:	Global health, Public health
Keywords:	PUBLIC HEALTH, Maternal medicine < OBSTETRICS, EPIDEMIOLOGY

SCHOLARONE™
Manuscripts

I, the Submitting Author has the right to grant and does grant on behalf of all authors of the Work (as defined in the below author licence), an exclusive licence and/or a non-exclusive licence for contributions from authors who are: i) UK Crown employees; ii) where BMJ has agreed a CC-BY licence shall apply, and/or iii) in accordance with the terms applicable for US Federal Government officers or employees acting as part of their official duties; on a worldwide, perpetual, irrevocable, royalty-free basis to BMJ Publishing Group Ltd ("BMJ") its licensees and where the relevant Journal is co-owned by BMJ to the co-owners of the Journal, to publish the Work in this journal and any other BMJ products and to exploit all rights, as set out in our licence.

The Submitting Author accepts and understands that any supply made under these terms is made by BMJ to the Submitting Author unless you are acting as an employee on behalf of your employer or a postgraduate student of an affiliated institution which is paying any applicable article publishing charge ("APC") for Open Access articles. Where the Submitting Author wishes to make the Work available on an Open Access basis (and intends to pay the relevant APC), the terms of reuse of such Open Access shall be governed by a Creative Commons licence – details of these licences and which Creative Commons licence will apply to this Work are set out in our licence referred to above.

Other than as permitted in any relevant BMJ Author's Self Archiving Policies, I confirm this Work has not been accepted for publication elsewhere, is not being considered for publication elsewhere and does not duplicate material already published. I confirm all authors consent to publication of this Work and authorise the granting of this licence.

For peer review only

Intended versus actual delivery location and factors associated with change in delivery location among pregnant women in Southern Province, Zambia: a pre-specified secondary observational analysis of the ZamCAT trial

Hiwote Solomon[1], Elizabeth G Henry[2,3], Julie M Herlihy[4], Kojo Yeboah-Antwi[5], Godfrey Biemba[1,6], Kebby Musokotwane[7], Afsah Bhutta[8], Davidson H. Hamer[3,9], Katherine E.A. Semrau[10,11,12]

[1] Boston University School of Public Health, Boston, Massachusetts, USA
[2] Department of Global Health and Population, Harvard TH Chan School of Public Health, Boston, Massachusetts, USA
[3] Department of Global Health, Boston University School of Public Health, Boston, Massachusetts, USA
[4] Department of Pediatrics, Boston University School of Medicine, Boston, Massachusetts, USA
[5] Fr. Thomas Alan Rooney Memorial Hospital, Asankrangwa, Western Region, Ghana
[6] Zambia National Health Research Authority, Lusaka, Zambia
[7] Levy Mwanawasa University Teaching Hospital, Lusaka, Zambia
[8] Dow University of Health Sciences, Karachi, Pakistan1 Section of Infectious Diseases,
[9] Department of Medicine, Boston University School of Medicine, Boston, MA, USA
[10] Ariadne Labs, Brigham and Women's Hospital and Harvard TH Chan School of Public Health, Boston, Massachusetts, USA
[11] Division of Global Health Equity & Department of Medicine, Brigham and Women's Hospital, Boston, Massachusetts, USA
[12] Department of Medicine, Harvard Medical School, Boston, Massachusetts, USA

Corresponding author
Hiwote Solomon, MPH
Boston University School of Public Health
801 Massachusetts Avenue
Boston, MA 02119
solomohi@bu.edu

For peer review only

Abstract

Objectives: This pre-specified, secondary analysis of the Zambia Chlorhexidine Application Trial (ZamCAT) aimed to determine the proportion of women who did not deliver where they intended, to understand the underlying reasons for the discordance between planned and actual delivery locations; and to assess sociodemographic characteristics associated with concordance of intention and practice.

Design: Pre-specified, secondary analysis from randomized controlled trial

Setting: Recruitment occurred in 90 primary health facilities (HFs) with follow-up in the community in Southern Province, Zambia

Participants: Between Feb 15, 2011, and Jan 30, 2013, 39,679 pregnant women enrolled in ZamCAT.

Secondary outcome measures: The location where mothers gave birth (home vs. HF) was compared to their planned delivery location.

Results: When interviewed antepartum, 92% of respondents intended to deliver at a HF, 6.1% at home, and 1.2% had no plan. However, of those who intended to deliver at a HF, 61% did; of those who intended to deliver at home, only 4% did; and of those who intended to deliver at home, 2% delivered instead at a HF. Among women who delivered at home, women who were aged 25-34 and $\geq$ 35 years were more likely to deliver where they intended than women aged 20-24 years (aOR=1.31, 95% CI: 1.14, 1.50,) and (aOR=1.32, 95% CI: 1.12, 1.57), respectively. Women who delivered at HFs had greater odds of delivering where they intended if they received any primary schooling (aOR=1.34, 95% CI=1.09, 1.72) or more than a primary school education (aOR=1.54, 95% CI=1.17, 2.02), were literate (aOR=1.30, 95% CI=1.09, 1.54), and were not in the lowest quintile of the wealth index.

For peer review only

Conclusion: Discrepancies between intended and actual delivery locations highlight the need to go beyond the development of birth plans and exposure to birth planning messaging. More research is required to address barriers to achieving intentions of a facility-based childbirth.

Trial Registration: ClinicalTrials.gov Identifier: NCT01241318

Funding Statement: This secondary analysis research received no specific grant from any funding agency in the public, commercial or not-for-profit sectors. The original data collection and primary trial was funded by the Bill & Melinda Gates Foundation (global health grant number OPPGH5298). The findings and conclusions contained within are those of the authors and do not necessarily reflect positions or policies of the Bill & Melinda Gates Foundation.

Word Count: Abstract: 300; Manuscript: 3,625

For peer review only

Article Summary

Strengths and limitations of this study:

- In this large, cluster-randomized controlled trial, we were able to gain information on >39,000 pregnant women enrolled with less than 5% loss to follow-up, providing robust estimates of factors related to birth location intentions and actual practice.
- The study was further strengthened by variation in intended location of birth as well as differences in actual practice of delivery location.
- Social desirability bias among respondents as questions such as birth plan and reasons behind delivery choice may have influenced women to respond that they intended to deliver in a facility.
- Qualitative data would be helpful to explore the underlying motivations and decision-making processes around intention and actual delivery locations.

For peer review only

Introduction

Each year, 140 million newborns are born worldwide.[1] While there has been global progress in reducing child mortality, corresponding rates of reduction in newborn mortality have been far slower and annually 2.5 million newborn deaths occur.[2] As the world strives to meet the Sustainable Development Goals (SDGs) by 2030, it is important to understand not only the direct causes, but also health systems barriers and social determinants of health that lead to poor maternal and newborn outcomes.[3] Factors responsible for these outcomes include poverty, illiteracy, issues of women's empowerment and systematic marginalization of communities on the basis of gender, ethnicity or geography.[2,4] In sub-Saharan Africa, birth planning and facility-based childbirth have been core components of interventions to improve maternal and neonatal outcomes. However, this has been insufficient as health systems need to be structurally redesigned to respond to local contexts.[5] Given the emphasis on birth planning in developing countries, it is important to understand why women deliver in locations that differ from what they intended, and what contributes to that change in plan, in order to adequately design interventions and messaging to address specific barriers that prevent women from delivering where they intended.[6–8]

Health facility delivery at primary health facilities with support from referral-level facilities for complicated births has been shown to improve maternal and neonatal outcomes.[5,7,9–11] In order to better understand some of the underlying factors and determinants of access to health facilities equipped with emergency obstetrical and newborn care (EmONC) and skilled birth attendants in rural Zambia, we used data from the Zambia Chlorhexidine Application Trial (ZamCAT), a cluster randomized controlled trial that assessed whether daily 4% chlorohexidine cord cleansing was more effective than dry cord care for prevention of neonatal deaths and omphalitis (umbilical cord infection) in Southern Province.[12] The aim of this pre-specified, secondary analysis was to

For peer review only - http://bmjopen.bmj.com/site/about/guidelines.xhtml

determine the proportion of women who did not deliver where they had intended, to understand the underlying reasons for the discordance between planned and actual delivery locations; and to determine sociodemographic characteristics associated with where pregnant women intended to deliver [i.e. concordance with intention and practice].

Methods

Study Site:

Southern Province, one of ten provinces in Zambia, consists of 13 districts with an estimated population of 2.1 million in 2020.[13] At the time of the ZamCAT study, the 2013-14 Zambia Demographic Health Survey (ZDHS) reported that nearly all women (96%) received at least one antenatal care visit during pregnancy.[14] Additionally, more than half of women (55.9%) in Southern Province delivered in a health facility (predominately public sector facilities), while 41% delivered at home.[14] Skilled birth attendants were present for 55% of all deliveries in Southern Province, while untrained family or relatives assisted with 22% of home deliveries.[14]

Study Design

ZamCAT, conducted from 2011-2013, was a cluster-randomized trial that enrolled and followed a cohort of 39,679 pregnant women living in six districts of Southern Province from before delivery to 28 days after delivery (ClinicalTrials.gov Identifier: NCT01241318). The analysis presented here was an *a priori* research question focusing on one of the ZamCAT study's secondary outcomes, namely: where did pregnant women enrolled in ZamCAT plan to deliver, what were their reasons for their intended location, and what was their actual practice? Our analysis also assessed factors that may have influenced intended and actual delivery locations.

For peer review only - http://bmjopen.bmj.com/site/about/guidelines.xhtml

In the main trial, the units of randomization (clusters) for ZamCAT were Zambian government-run or mission-run primary health-care centers in Southern Province. Ninety clusters were randomized into two groups: (1) clean dry cord care in accordance with Ministry of Health guidelines (control) and (2) daily 4% chlorhexidine cord washes until three days after the umbilical cord fell off (intervention). The study location included a wide range of health facilities, both urban and rural, with differences in staffing and catchment area population characteristics.

Eligible women were in their second or third trimester, aged 15 years or older, planning to stay in the catchment area until 28 days postpartum, and willing to provide informed consent and complete cord care as per cluster assignment.[12] Women were screened by field monitors (data collectors), and women were enrolled either at a facility-based antenatal care visit or through community outreach events. At enrollment, a detailed questionnaire was administered which included questions on household information, birth plan, and antenatal care characteristics. After the enrollment survey was completed, participants were encouraged to deliver at the nearest health facility and were given standard newborn care messages which included information about delivery location, breastfeeding, cord care and danger signs of ill health in their newborn baby as per national and Ministry of Health guidelines. At a home visit two weeks after enrollment, all participants, irrespective of study allocation, were provided a standard clean delivery kit. A birth notification system was developed in collaboration with the community specific to each cluster to alert study team of births.[15] For both study arms, field monitors conducted routine home visits at least once during the antenatal period and four times after delivery (days 1, 4, 10, and 28 postpartum). Detailed information on where the woman delivered and why was collected during the day 4 postpartum visit. Data were collected using paper forms designed in the Teleforms

For peer review only - http://bmjopen.bmj.com/site/about/guidelines.xhtml

system (HP Cambridge, UK). Supervisors reviewed forms for illegible and missing entries and worked with the field monitors to make sure they were complete. The forms were transported to the central study office in Choma where a data management team scanned and entered them into a Microsoft Access Database.

Statistical analysis

Using the full cohort of women enrolled in the study, we assessed women's socioeconomic and pregnancy-related characteristics, and then related them to the outcome of interest (delivery where intended). Our primary outcome was a four-level categorical variable: intended to deliver at health facility and did, intended to deliver at home and did, intended to deliver at a health facility but delivered at home, and intended to deliver at home but delivered at a health facility. Socioeconomic and pregnancy-related characteristics were documented during the enrollment visit and compared across the intention/practice groups. Pregnancy-related characteristics potentially associated with the outcomes were compared between the two groups using the $\chi 2$ test, Fisher's exact test, t-tests, or non-parametric Wilcoxon rank sum tests, as appropriate for continuous or categorical variables. We also assessed the reasons women indicated for why they intended to deliver at a certain location and reasons given after actual delivery on where their actual birth location; women could choose among eight different reasons for why they chose their intended or actual delivery location. Women were allowed to choose more than one reason for why they chose their intended or actual delivery location. All statistical analyses were completed using SAS University Edition®.[16] We assessed the association of sociodemographic characteristics with women delivering where they intended to deliver or elsewhere using logistic regression modeling. We also conducted a stratified

For peer review only

For peer review only - http://bmjopen.bmj.com/site/about/guidelines.xhtml

logistic regression model to assess the predictors of women delivering where they intended stratified by actual delivery location (home and facility).

The Boston University Medical Campus Institutional Review Board (FWA# 00008404) and University of Zambia Research Ethics Committee (FWA# 00001131) provided ethical clearance. The Zambia National Health Research Authority approved the study. All women provided written informed consent, which was obtained in English or Tonga.

Patient and Public Involvement

Study preparations for the overall ZamCAT trial required baseline formative ethnographic research, substantial community sensitization, and engagement with three levels of stakeholders, each necessitating different strategies. The process of community engagement for ZamCAT is detailed further by Hamer et al.[15] Community leaders and neighborhood health committees in the 90 clusters identified appropriate Field Monitor (data collector) candidates according to specific selection criteria. The local health facility staff assisted by ranking lists of potential data collectors provided by the community. Further, the effective birth notification system was developed by the community leaders. Results from the primary study were shared through community meetings in collaboration with the district health offices and at the Zambian National Health Research Conference.

Results

A total of 39,679 pregnant women were enrolled in ZamCAT between Feb 15, 2011 and Jan 30, 2013.[12] During the study 38,131 women had 38,579 deliveries; 1,548 enrolled women were excluded as a result of false pregnancies, miscarriages or abortion, withdrawal from study before

For peer review only - http://bmjopen.bmj.com/site/about/guidelines.xhtml

delivery, lost to follow-up, or death. We had intended and actual delivery information on 37,105 of the enrolled population. The mean age (± standard deviation [SD]) of the women was 25.6 ± 6.9 years, 10% had no formal education, and 52% had only a primary education (Table 1). Of the 37,105 with birth plans stated, 467 (1.2%) had no plan, 2,278 (6.1%) planned to deliver at home, 33,953 (91.5%) intended to deliver at a health facility, and 407 (1.1%) stated other. In practice, 13,139 (35.4%) actually delivered at home, 23,666 (63.7%) actually delivered at a health facility, and 300 (0.8%) delivered in another location.

Table 1. Demographic, household, and pregnancy characteristics of women enrolled in ZamCAT

**Characteristics**	Intended to deliver at health facility and did **n=22 651**	Intended to deliver at home and did **n=1515**	Intended to deliver at health facility but delivered at home **n=11 016**	Intended to deliver at home but delivered at a health facility **n=755**
Maternal Age (years), n (%)				
<20	5919 (26.1%)	185 (12.2%)	2115(19.2%)	215 (28.5%)
20-35	14473 (63.9%)	1114 (73.5%)	7626 (69.2%)	442 (58.5%)
>35	2259 (10.0%)	216 (14.3%)	1275 (11.6%)	98 (13.0%)
Missing				
Tribe, n (%)				
Tonga	19 295 (85.2%)	1431 (94.5%)	10 257 (93.1%)	670 (88.7%)
Ila	142 (0.6%)	7 (0.5%)	53 (0.5%)	4 (0.5%)
Lozi	1175 (5.2%)	20 (1.3%)	238 (2.2%)	28 (3.7%)
Nyanja	687 (3.0%)	18 (1.2%)	147 (1.3%)	13 (1.7%)
Bemba	568 (2.5%)	23 (1.5%)	135 (1.2%)	24 (3.2%)
Other	781 (3.4%)	15 (1.0%)	183 (1.7%)	12 (1.6%)
Missing	3 (0.08%)	1 (0.1%)	3 (0.03%)	4 (0.5%)
Women's Education Level, n (%)				
No education	1681 (7.4%)	265 (17.5%)	1442 (13.1%)	95 (12.6%)
Some primary education	10 779 (47.6%)	1001 (66.1%)	6487 (58.9%)	366 (48.5%)
Some secondary education	9884 (43.6%)	246 (16.2%)	3067 (27.8%)	290 (38.4%)
Higher than secondary education	288 (1.3%)	2 (0.13%)	14 (0.1%)	0 (0.0%)
Unknown or Missing	19 (0.1%)	1 (0.1%)	6 (0.06%)	4 (0.5%)
Maternal Literacy, n (%)				
Illiterate	4875 (21.5%)	611 (40.3%)	3720 (33.8%)	226 (29.9%)
Can read a bit	10620 (46.9%)	716 (47.3%)	5212 (47.4%)	332 (44.0%)
Can read very well	7078 (31.2%)	177 (11.7%)	2022 (18.4%)	191 (25.3%)
No response or Missing	78 (0.3%)	11 (0.7%)	62 (0.6%)	6 (0.8%)
Wealth Index, n (%)				
1-lowest	5214 (23.0%)	541 (35.7%)	3154 (28.6%)	246 (32.6%)
2	5407 (23.9%)	421 (27.8%)	2910 (26.4%)	175 (23.2%)
3	5490 (24.2%)	361 (23.8%)	2807 (25.5%)	181 (24.0%)
4-highest	6540 (28.9%)	192 (12.7%)	2145 (19.5%)	151 (20.0%)

For peer review only - http://bmjopen.bmj.com/site/about/guidelines.xhtml

Marital Status, n (%)				
Single	4173 (18.4%)	82 (5.4%)	1243 (11.3%)	132 (17.5%)
Married or Cohabitating	18 267 (80.7%)	1405 (92.7%)	9644 (87.6%)	600 (79.5%)
Separated, Divorced, or Widowed	204 (0.9%)	26 (1.7%)	128 (1.2%)	19 (2.5%)
Missing	7 (0.03%)	2 (0.1%)	1 (0.01%)	4 (0.5%)
Median household size (IQR)	6 (4-8)	6 (4-8)	6 (4-8)	6 (4-8)
Time walking from home to health facility				
<1h	9276 (41.0%)	293 (19.3%)	2301 (20.9%)	253 (33.5%)
1 to <2 h	7823 (34.5%)	526 (34.7%)	4202 (38.1%)	278 (36.8%)
2 to <3h	3981 (17.6%)	449 (29.6%)	3054 (27.7%)	140 (18.5%)
3 to <4 h	1047 (4.6%)	167 (11.0%)	1017 (9.2%)	58 (7.7%)
4 to <5 h	250 (1.1%)	43 (2.8%)	248 (2.3%)	12 (1.6%)
5 h or more	120 (0.5%)	12 (0.8%)	129 (1.2%)	8 (1.1%)
Unknown	81 (0.4%)	17 (1.1%)	42 (0.4%)	2 (0.3%)
Missing	74 (0.3%)	8 (0.5%)	23 (0.2%)	4 (0.5%)
**Pregnancy Characteristics**				
Parity, mean (SD)	2.7 (2.3)	2.4 (2.3)	2.3 (2.3)	2.5 (2.3)
Primiparous, n (%)	4230 (18.7%)	231 (15.3%)	2188 (19.9%)	119 (15.8%)
Multiparous, n (%)	11 939 (52.7%)	1131 (74.7%)	7086 (64.3%)	401 (53.1%)
Missing, n (%)	6482 (28.6%)	153 (10.1%)	1742 (15.8%)	235 (31.1%)
Gravida, mean (SD)	3.8 (2.4)	3.5 (2.3)	3.4 (2.3)	3.5 (2.3)
Primigravida, n (%)	6327 (27.9%)	148 (9.8%)	1702 (15.5%)	225 (29.8%)
Multigravida, n (%)	16 294 (71.4%)	1362 (89.9%)	9298 (84.4%)	525 (69.5%)
Missing, n (%)	30 (0.1%)	5 (0.3%)	16 (0.2%)	5 (0.7%)
Gestational age at enrollment, mean (SD)	27.9 (6.8)	28.0 (7.0)	28.1 (7.1)	28.1 (7.0)
Time between water breaking and delivery, n (%)				
Less than 24 hours	20,259 (91.7%)	1344 (88.7%)	10,123 (91.9%)	418 (55.4)
24-48 hours	781 (3.5%)	25 (1.7%)	168 (1.5%)	35 (4.6%)
More than 48 hours	120 (0.54%)	8 (0.53%)	26 (0.2%)	2 (0.3%)
Don't know	915 (4.1%)	120 (8.0%)	446 (4.1%)	32 (4.2%)
Missing	576 (2.5%)	1 (1.2%)	253 (2.3%)	268 (35.5%)
*Those who responded to having "no plan" or "other" when asked about intended delivery location, and those who responded as "other" when asked about actual delivery location were excluded from demographic analysis (n=1,168).				

Reasons for planned vs. actual delivery location

At study enrollment, respondents were asked about their birth plan and where they planned to deliver as well as why (Table 2). Almost all women had a delivery location in their plan (98.8%) and nearly all women (91.6%) reported that they planned to deliver in a health facility. Women who planned to deliver in a health facility stated they were motivated by safety for mother/baby

(83.9%) and need for skilled attendance (81.3%). Among those who intended to deliver at home, distance was the most commonly noted reason (37.9%) and same location as previous deliveries (29.2%).

Table 2. Planned delivery location and rationale

	No Plan (N=467)		Health Facility (N=33,953)		Home (N=2,278)		Other (N=407)	
	n	%	n	%	n	%	n	%
Same location as prior delivery	3	0.6%	4,237	12.5%	666	29.2%	55	13.5%
Need for skilled attendance	14	3.0%	27,578	81.2%	402	17.7%	281	69.0%
Financial restraints	13	2.8%	1,058	3.1%	443	19.5%	16	3.9%
Distance	10	2.1%	4,714	13.9%	851	37.4%	45	11.1%
Relationship with Providers	3	0.8%	453	1.3%	175	7.7%	8	2.0%
Family/social expectations	2	0.5%	714	2.1%	264	11.6%	2	0.5%
Safety for mother/baby	12	2.6%	28,548	84.1%	464	20.4%	293	72.0%
Other	3	0.6%	276	0.8%	47	2.1%	33	8.1%
Missing	0	0.0%	1	0.0%	5	0.2%	4	1.0%

**Note "Other" in birth plan often includes HOSPITAL delivery which is separate from health facility delivery as well as TBA delivery.*

After delivery, women were asked about where they actually delivered and why (Table 3). Although nearly all women indicated that they planned to deliver at a health facility, only 63.8% (N=23,666) actually did. The primary motivations for delivery in the facility was need for skilled attendance (54.6%) and safety for the mother and baby (55.1%). While only 6% of women intended to deliver at home, over one-third (35.4%) of women actually did (N=13,139). The top three reasons for delivering at home were distance (29.7%), need for skilled attendance (43.0%), and safety for mother/baby (44.7%).

For peer review only - http://bmjopen.bmj.com/site/about/guidelines.xhtml

Table 3. Actual delivery location and reasoning

	Health Facility (N=23,666)		Home (N=13,139)		Other (N=300)	
	n	%	n	%	n	%
Same location as prior delivery	3,182	13.5%	2,062	15.7%	47	15.7%
Need for skilled attendance	12,916	54.6%	5,654	43.0%	164	54.7%
Financial restraints	1799	7.6%	2,234	9.4%	13	4.3%
Distance	6,303	26.6%	3,898	29.7%	74	24.7%
Relationship with Providers	571	2.4%	426	3.2%	9	3.0%
Family/social expectations	1,251	5.3%	961	7.5%	17	5.7%
Safety for mother/baby	13,048	55.1%	5,866	44.7%	170	56.7%
Other	1,468	6.2%	792	6.0%	23	7.7%
Missing	1,353	5.7%	762	5.8%	15	5.0%

Note: Health facility in actual delivery location includes hospital delivery

Intended vs. actual delivery location

Overall, 67% of women delivered in their intended location. Almost two-thirds of women (n=22,651, 61.1%) who intended to deliver at a health facility actually did (concordant); in contrast, almost one-third of women who intended to deliver at a health facility (n=11,016, 29.7%) delivered at home (discordant). Of those who intended to deliver at home, only 4.1% (n=1515) actually did. Additionally, of those women who intended to deliver at home, 2.0% (n=755) delivered instead at a health facility. Excluded were those who indicated “no plan” or “other” for intended delivery location and those who indicated “other” for actual delivery location.

We observed that 18.7% of those who intended to deliver at a heath facility and did were primiparous, compared to women who intended to deliver at a health facility but delivered at home (19.9%). For those women who were multiparous, more than half (52.7%) intended to deliver at home and actually did; two-thirds (64.3%) of women who intended to deliver at a health facility, delivered at home. We found that time between water breaking and delivering in less than 24 hours was not shorter for those who intended to delivery at a health facility but delivered at home.

For peer review only - http://bmjopen.bmj.com/site/about/guidelines.xhtml

Women who intended to deliver at home and subsequently delivered at a health center stated they needed a skilled attendant for the safety of the mother and baby (Table 4). About 30% of respondents, who indicated distance as a reason for delivering where they did, delivered at home. For those who intended to deliver at a health facility and did, 41% lived less than a one hour walk from the health facility. Of women who intended to deliver at home and did, 64% lived between one to three hours walk from the nearest health facility. Among those who intended to deliver at a health facility and actually delivered at home, 46.3% indicated that they did so because of safety for mother/baby. Additionally, this same group indicated a need for skilled attendance as a reason for delivering at home versus at a health facility. Among those who intended to and actually delivered at home, only 8.9% indicated family/social expectations as a reason while 34.5% indicated safety for mother/baby as a reason.

Table 4. Comparison of Intended and Actual Delivery Location, with Reasons for Actual Delivery Location

Reasons for Actual Delivery Location	Intended to deliver at health facility and did (N=22 651)		Intended to deliver at home and did (N=1515)		Intended to deliver at health facility but delivered at home (N=11 016)		Intended to deliver at home but delivered at a health facility (N=755)	
	**n**	**%**	**n**	**%**	**n**	**%**	**n**	**%**
Same location as prior delivery	3020	13.4%	279	18.4%	1677	15.2%	120	15.9%
Need for skilled attendance	12458	54.5%	476	31.4%	4947	44.9%	353	46.8%
Financial restraints	1708	7.7%	149	9.8%	1021	9.3%	72	9.5%
Distance	5987	26.6%	505	33.3%	3229	29.3%	233	30.9%
Relationship with Providers	528	2.4%	76	5.0%	326	3.0%	34	4.5%
Family/social expectations	1182	5.3%	135	8.9%	782	7.1%	48	6.4%
Safety for mother/baby	12585	36.9%	523	34.5%	5095	46.3%	332	44.0%
Other	1408	6.2%	107	7.1%	654	5.9%	44	5.8%
Missing	1296	6.2%	89	5.9%	628	5.7%	47	6.2%
**Time walking from home to health facility**								
<1h	9276	41.0%	293	19.3%	2301	20.9%	253	33.5%
1 to <2 h	7823	34.5%	526	34.7%	4202	38.1%	278	36.8%
2 to <3h	3981	17.6%	449	29.6%	3054	27.7%	140	18.5%
3 to <4 h	1047	4.6%	167	11.0%	1017	9.2%	58	7.7%

For peer review only - http://bmjopen.bmj.com/site/about/guidelines.xhtml

4 to <5 h	250	1.1%	43	2.8%	248	2.3%	12	1.6%
5 h or more	120	0.5%	12	0.8%	129	1.2%	8	1.1%
Unknown	81	0.4%	17	1.1%	42	0.4%	2	0.3%
Missing	74	0.3%	8	0.5%	23	0.2%	4	0.5%

Predictors of women delivering where intended

Table 5 shows the result of our stratified logistic regression model where we assessed predictors of women delivering where they intended to, stratified by home delivery and facility delivery. Among women who delivered at home, we found that women who were aged 25-34 and ≥ 35 years were more likely to deliver where they intended than women aged 20-24 years (aOR=1.31, (95% CI=1.14, 1.50), p=0.17) and (aOR=1.32, (95% CI=1.12, 1.57), p=0.003), respectively. Women who were 15-19 years and delivered at home had lower odds (aOR=0.77, (95% CI=0.60, 0.93), p<.0001) of delivering where they intended compared to those aged 20-24 years. Women who delivered at home and had more than a primary education were less likely to deliver where they intended when compared to those with no education (aOR=0.55, (95% CI=0.43, 0.69), p<0.0001). Those who delivered at home and were not in the lowest wealth quintile were less likely to deliver where they intended (Table 5). When examining the time to walk to nearest health facility, we found that those who delivered at home had higher odds of delivering where they intended to if they lived two to less than three hours from nearest health facility (aOR=1.16, (95% CI=1.02, 1.33), p=0.03) or three to less than four hours away from nearest health facility (aOR=1.27, (95% CI=1.05, 1.54), p=0.01).

Women who delivered at the facility had greater odds of delivering where they intended if they received any primary schooling (aOR=1.34, (95% CI=1.09, 1.72), p=0.008) or more than a primary school education (aOR=1.54, (95% CI=1.17, 2.02), p=0.002), were literate (aOR=1.30 (95%

For peer review only - http://bmjopen.bmj.com/site/about/guidelines.xhtml

CI=1.09, 1.54), p=0.003), were in the second (aOR=1.37, (95% CI=1.16, 1.63), p=0.0003), third (aOR=1.34, (95% CI: (1.13, 1.58), p=0.0007), or fourth (aOR=1.69, (95% CI=1.40, 2.04), p<0.0001) quintile of the wealth index, and those who experienced headache as a complication during pregnancy (aOR=1.71, (95% CI=1.12, 2.60), p=0.01). In contrast, women who delivered at health facility had lower odds of delivering where they intended if it was their first pregnancy (aOR=0.67, (95% CI=0.55, 0.80), p<0.0001) compared to those who had been pregnant before, lived between a three to less than four hour walk from the nearest health facility (aOR=0.74, (95% CI=0.57, 0.96), p=0.02) compared to those who lived a one to less than two hour walk, lapsed 24-48 hours between water breaking and delivery (aOR=0.534 (95% CI=0.40, 0.73), p<0.0001) compared to those who only lapsed less than 24 hours, and those who experienced bleeding (aOR=0.60, (95% CI=0.40, 0.90), p=0.01), fits (aOR=0.40, (95% CI=0.29, 0.85), p=0.02), vaginal discharge (aOR=0.24, (95% CI=0.13, 0.42), p<.0001), and/or heavy bleeding (aOR=0.73, (95% CI=0.56, 0.97), p=0.03) compared to those who did not experience these symptoms.

For peer review only - http://bmjopen.bmj.com/site/about/guidelines.xhtml

Table 5. Predictors of women delivering where they intended to, stratified by delivery location

Characteristics	Among those that delivered at home, adjusted odds ratio (aOR) of delivering where intended		Among those that delivered at facility, adjusted odds ratio (aOR) of delivering where intended	
	**aOR (95% CI)**	**p-value**	**aOR (95% CI)**	**p-value**
Maternal Age (years)				
15-19	**0.77 (0.64 0.93)**	**0.007**	**0.69 (0.57, 0.82)**	**<.0001**
20-24	ref		ref	
25-34	**1.31 (1.14, 1.50)**	**<.0001**	0.89 (0.75, 1.05)	**0.17**
>35	**1.32 (1.12, 1.57)**	**0.001**	**0.73 (0.59, 0.90)**	**0.003**
Women's Education Level				
No education	ref		ref	
Any primary	0.90 (0.75, 1.06)	0.21	**1.34 (1.09, 1.72)**	**0.008**
More than primary	**0.55 (0.43, 0.69)**	**<.0001**	**1.54 (1.17, 2.02)**	**0.002**
Marital Status				
Married	ref		ref	
Not married	**0.67 (0.55, 0.83)**	**0.0002**	0.89 (0.76, 1.06)	0.18
Can read				
No	ref		ref	
Yes	1.05 (0.91, 1.20)	0.50	**1.30 (1.09, 1.54)**	**0.003**
Household Light Source				
No electricity	ref		ref	
Electricity	0.81 (0.57, 1.16)	0.25	1.27 (0.95, 1.69)	0.11
Wealth Index				
1-lowest	**ref**	**ref**	**ref**	**ref**
2	**0.85 (0.74, 0.97)**	**0.02**	**1.37 (1.16, 1.63)**	**0.0003**
3	**0.76 (0.66, 0.88)**	**0.0002**	**1.34 (1.13, 1.58)**	**0.0007**
4-highest	**0.56 (0.46, 0.67)**	**<.0001**	**1.69 (1.40, 2.04)**	**<0.0001**
Primigravida				
No	ref		ref	
Yes	0.90 (0.73, 1.11)	0.33	**0.67 (0.55, 0.80)**	**<0.0001**
Time to walk to nearest health facility				
<1h	1.10 (0.94, 1.28)	0.24	1.14 (0.98, 1.32)	0.10
1 to <2 h	ref		ref	ref
2 to <3h	**1.16 (1.02, 1.33)**	**0.03**	0.92 (0.78, 1.09)	0.34
3 to <4 h	**1.27 (1.05, 1.54)**	**0.01**	**0.74 (0.57, 0.96)**	**0.02**
4 to <5 h	1.20 (0.84, 1.68)	0.32	0.80 (0.48, 1.35)	0.41
5 h or more	0.68 (0.37, 1.24)	0.20	0.70 (0.34, 1.45)	0.34
Time between water breaking and delivery				
Less than 24 hours	**ref**		ref	
24-48 hours	1.04 (0.68, 1.59)	0.87	**0.54 (0.40, 0.73)**	**<0.0001**
More than 48 hours	2.11 (0.95, 4.69)	0.07	1.34 (0.44, 4.38)	0.58
Don't know	**1.93 (1.56, 2.38)**	**<.0001**	**0.49 (0.37, 0.64)**	**<0.0001**
Complications during pregnancy*				

High fever	1.45 (1.00, 2.11)	0.05	0.79 (0.54, 1.16)	0.23
Bleeding	1.23 (0.80, 2.05)	0.31	**0.60 (0.40, 0.90)**	**0.01**
Fits	2.15 (0.79, 5.82)	0.13	**0.40 (0.19, 0.85)**	**0.02**
Headache	1.07 (0.79, 1.44)	0.67	**1.71 (1.12, 2.60)**	**0.01**
Vaginal discharge	0.50 (0.15, 1.61)	0.24	**0.24 (0.13, 0.42)**	**<0.0001**
Heavy bleeding	1.08 (0.78, 1.50)	0.63	**0.73 (0.56, 0.97)**	**0.03**
Swelling	0.88 (0.63, 1.22)	0.43	0.82 (0.62, 1.10)	0.19

* Note: Referent groups for complications were the absence of specified complications
**Adjusted for maternal age, women's education, marital status, literacy, wealth index, housing light source

Discussion

Overall, birth plans were widely prepared in this cohort and >90% of women indicated a plan to deliver at a health facility, consistent with the 2013-2014 Zambia Demographic Health Survey (ZDHS). [14] However, only two-thirds of women had actual delivery location in line with their birth plan and >35% of women delivered at home. Our analysis demonstrates clear deviations from the birth plan during the actual delivery, especially among those who had expressed an intent to deliver at a facility. Discrepancy between planning and actual delivery behavior highlights that the development of birth plans and exposure to birth planning messaging are insufficient to address barriers to adhering to plans to have a facility-based childbirth.

Several reasons may underlie the discrepancy between intended location and actual practice around childbirth. One potential reason could be due to women's increased concerns about safety at facilities that are poorer quality. Interestingly, 46% of women in our sample indicated safety for mother/baby as a reason for why they delivered at home even though they intended to deliver at a health facility. Unfortunately, we do not know if this was driven by fear of poor treatment at the facility or fear of transporting/moving the laboring women. In some instances, poorly functioning health systems are unable to engage women in antenatal and post-natal care to create sufficient continuity of care.[17] Some of these barriers may relate to a sense of fatalism around maternal and

For peer review only

newborn care, and are important contributors to preventable mortality.[18] Such disparities present a major challenge to policies and programs aiming to mitigate the burden of maternal and neonatal mortality. The large proportion of facility births in unclean, unhygienic environments, and poor postnatal care may be important contributors to why women indicate safety for mother/baby as a reason why they changed their birth plan from intending to deliver at a health facility to delivering at home.

Another factor that may contribute to discrepancy between intention and practice is the dynamic between provider and patient.[19–21] Among those who intended to deliver at a health facility but instead delivered at home, 45% indicated need for skilled delivery as a reason. While this seems counterintuitive, one reason for this may be the presence of traditional birth attendants (TBAs) in the community who women regard as skilled attendants.[22,23] Although a policy change in Zambia resulted in TBAs no longer being trained and recommended that they stop conducting deliveries at home, a study on reasons for home delivery and use of TBAs in rural Zambia by Sialubanje et al. found that most women had positive attitudes towards TBAs and regarded them as respectful, skilled, and trustworthy.[19] Moreover, most women indicated that negative attitudes toward the quality of services at the clinic as a reason for why they delivered at home.[19] Smith et al. focused on behavioral drivers in labor and delivery wards in Chipata District in Eastern Province, Zambia and found that women in the wards are often subjected to disrespect and abuse.[24] Another study in rural Kenya found that although the community was aware of the risks of delivering with a TBA, many still preferred to deliver with a TBA due to their availability, accessibility, and their friendly attitude.[22] In Zambia, a study by Biemba et al. which utilized data from the ZamCAT study, found that TBAs often end up tending to deliveries even in health facilities due to a lack of qualified

For peer review only - http://bmjopen.bmj.com/site/about/guidelines.xhtml

personnel.[25] As for the safety of mother and child, some women perceive delivering at the health facility as unsafe based on cultural values of elder women in the community.[26] Finally, in another study, women in Ethiopia who had low perceived susceptibility and severity were more likely to intend to deliver at home instead of at a health facility.[26,27]

Among women who intended to deliver at home distance was most often cited as a reason (37.9%), although this less often reported when women were asked why they delivered home post-delivery. Our data are similar to the 2013-14 ZDHS which reported that 32.2% of women in Southern Province indicated distance and lack of transportation as a reason for not delivering in a health facility. Additionally, a study in Ebonyi State, Nigeria on the choice of birth place among antenatal clinic attendees in rural mission hospitals also found that distance served as a major determinant of birth place location.[28] Distance as a barrier to accessing care is not uncommon in many resource-constrained countries in sub-Saharan Africa and Asia.[29,30] Scott el al. found that women who lived in remote areas of Zambia faced persistent challenges regarding delivery location.[31] The study which included four districts in Southern Province also found women who lived far from a health facility, attended fewer antenatal visits, were multigravida, and who did not utilize maternity waiting homes had higher odds of home delivery.[31] Maternal waiting homes have shown to increase likelihood of delivering at a health facility.[31–33] Given that most maternal waiting homes were renovated or built after 2013, our study did not collect data on how maternal waiting home influenced delivery locations for those further away from the health facility.

Our analysis is strengthened by the sample size and variation in adherence to intended delivery location. However, a key limitation is a likelihood for social desirability bias among respondents

For peer review only - http://bmjopen.bmj.com/site/about/guidelines.xhtml

as questions such as birth plan and reasons behind delivery choice may have influenced women to respond they intended to deliver in a facility. Despite the multiple messages provided to the participants during the antenatal period encouraging them to deliver in health facilities, a substantial number of women were unable or opted not to deliver at a facility. The decision on where to deliver may have been made in consultation with family members or influenced by other forces which were missed in our data capture. Another limitation is that the data are seven years old. Although our findings reflected facility-based childbirth trends around 2014,[14] facility-based childbirth in Zambia by 2018 had increased.[34] However, given the recent COVID-19 pandemic, facility-based childbirth has again decreased and our findings may be applicable.[34,35] Finally, we do not have qualitative data could have helped explore the underlying motivations around intention and actual delivery locations.

Understanding factors that influence women to change their birth plan as well as addressing structural and perceived barriers to facility delivery are important to improving care. Maternal and neonatal outcomes can be significantly improved if women delivered at a facility with at least basic emergency obstetric and newborn care.[36,37] Potential interventions that can address barriers to reaching facilities include provision of maternity waiting homes, addressing perceived safety and treatment of mothers and infants in facilities, supporting providers to provide high-quality/respectful maternity care, systemwide plans that support transport of pregnant women to a health facility, messaging around saving for transport to a health facility, further health education for those who are primigravida to deliver a health facility, and safe delivery incentive programs.

For peer review only - http://bmjopen.bmj.com/site/about/guidelines.xhtml

Contribution statement:

As the original co-investigators of the ZamCAT trial, DHH and KEAS were responsible for conceiving the original research question, designing the trial, and overseeing the study implementation. KS, DHH and HS were responsible for the conceptualization of this sub-analysis. HS was responsible for the methodology, conducting formal analysis, and the writing and revision of the manuscript. JMH, GB, KM and KY-A were part of the original research team for ZamCAT and were responsible for reviewing and editing the manuscript. EGH was responsible for data set creation; preliminary analysis and manuscript review. AB assisted with preliminary analysis, reviewing, and editing of the manuscript.

Competing interests: None declared.

Data Sharing Statement: The data that support the findings of this study are available from the corresponding author, upon reasonable request.

For peer review only

References

1. Graham W, Woodd S, Byass P, et al. Diversity and divergence: the dynamic burden of poor maternal health. *The Lancet*. 2016;388(10056):2164-2175.

2. UNICEF. Neonatal mortality. UNICEF DATA. Published 2018. Accessed May 13, 2020. https://data.unicef.org/topic/child-survival/neonatal-mortality/

3. United Nations. Transforming our world: the 2030 Agenda for Sustainable Development | Department of Economic and Social Affairs. Published 2015. Accessed January 9, 2021. https://sdgs.un.org/2030agenda

4. Liu L, Oza S, Hogan D, et al. Global, regional, and national causes of under-5 mortality in 2000–15: an updated systematic analysis with implications for the Sustainable Development Goals. *The Lancet*. 2016;388(10063):3027-3035.

5. Hanson C, Waiswa P, Pembe A, Sandall J, Schellenberg J. Health system redesign for equity in maternal and newborn health must be codesigned, country led, adapted to context and fit for purpose. *BMJ Glob Health*. 2020;5(10):e003748. doi:10.1136/bmjgh-2020-003748

6. McCauley H, Lowe K, Furtado N, Mangiaterra V, van den Broek N. What are the essential components of Antenatal Care? A systematic review of the literature and development of signal functions to guide monitoring and evaluation. *BJOG Int J Obstet Gynaecol*. Published online November 28, 2021. doi:10.1111/1471-0528.17029

7. Campbell OMR, Graham WJ, Lancet Maternal Survival Series steering group. Strategies for reducing maternal mortality: getting on with what works. *Lancet Lond Engl*. 2006;368(9543):1284-1299. doi:10.1016/S0140-6736(06)69381-1

8. Bhutta ZA, Das JK, Bahl R, et al. Can available interventions end preventable deaths in mothers, newborn babies, and stillbirths, and at what cost? *Lancet Lond Engl*. 2014;384(9940):347-370. doi:10.1016/S0140-6736(14)60792-3

9. Doctor HV, Nkhana-Salimu S, Abdulsalam-Anibilowo M. Health facility delivery in sub-Saharan Africa: successes, challenges, and implications for the 2030 development agenda. *BMC Public Health*. 2018;18(1):765.

10. Pervin J, Moran A, Rahman M, et al. Association of antenatal care with facility delivery and perinatal survival–a population-based study in Bangladesh. *BMC Pregnancy Childbirth*. 2012;12(1):111.

11. Roder-DeWan S, Nimako K, Twum-Danso NAY, Amatya A, Langer A, Kruk M. Health system redesign for maternal and newborn survival: rethinking care models to close the global equity gap. *BMJ Glob Health*. 2020;5(10):e002539. doi:10.1136/bmjgh-2020-002539

For peer review only

For peer review only - http://bmjopen.bmj.com/site/about/guidelines.xhtml

12. Semrau KEA, Herlihy J, Grogan C, et al. Effectiveness of 4% chlorhexidine umbilical cord care on neonatal mortality in Southern Province, Zambia (ZamCAT): a cluster-randomised controlled trial. *Lancet Glob Health*. 2016;4(11):e827-e836. doi:10.1016/S2214-109X(16)30215-7

13. Zambia Statistics Agency. Population and Demographic Projections, 2011-2035 - Zambia Data Portal. Published 2020. Accessed January 31, 2021. https://zambia.opendataforafrica.org/ZMPHC2015/population-and-demographic-projections-2011-2035

14. Central Statistical Office/Zambia, Ministry of Health/Zambia, University of Zambia Teaching Hospital Virology Laboratory, University of Zambia Department of Population Studies, Tropical Diseases Research Centre/Zambia, I. C. F. International. Zambia Demographic and Health Survey 2013-14. Published online March 1, 2015. Accessed February 14, 2020. https://dhsprogram.com/publications/publication-fr304-dhs-final-reports.cfm

15. Hamer DH, Herlihy JM, Musokotwane K, et al. Engagement of the community, traditional leaders, and public health system in the design and implementation of a large community-based, cluster-randomized trial of umbilical cord care in Zambia. *Am J Trop Med Hyg*. 2015;92(3):666-672.

16. SAS Institute. *SAS University Edition*.; 2020. Accessed January 9, 2021. https://support.sas.com/en/software/university-edition-support.html

17. Kyei-Nimakoh M, Carolan-Olah M, McCann TV. Access barriers to obstetric care at health facilities in sub-Saharan Africa—a systematic review. *Syst Rev*. 2017;6. doi:10.1186/s13643-017-0503-x

18. Sather M, Fajon AV, Zaentz R, Rubens CE. Global report on preterm birth and stillbirth (5 of 7): advocacy barriers and opportunities. *BMC Pregnancy Childbirth*. 2010;10(1):S5.

19. Sialubanje C, Massar K, Hamer DH, Ruiter RA. Reasons for home delivery and use of traditional birth attendants in rural Zambia: a qualitative study. *BMC Pregnancy Childbirth*. 2015;15(1):216. doi:10.1186/s12884-015-0652-7

20. Mannava P, Durrant K, Fisher J, Chersich M, Luchters S. Attitudes and behaviours of maternal health care providers in interactions with clients: a systematic review. *Glob Health*. 2015;11(1):36. doi:10.1186/s12992-015-0117-9

21. Yakong VN, Rush KL, Bassett-Smith J, Bottorff JL, Robinson C. Women's experiences of seeking reproductive health care in rural Ghana: challenges for maternal health service utilization. *J Adv Nurs*. 2010;66(11):2431-2441. doi:https://doi.org/10.1111/j.1365-2648.2010.05404.x

22. Cheptum JJ, Gitonga MM, Mutua EM, Mukui SJ, Ndambuki JM, Koima WJ. Perception about traditional birth attendants by men and women of reproductive age in rural Migori County, Kenya. *Int J Afr Nurs Sci*. 2017;7:55-61. doi:10.1016/j.ijans.2017.07.002

For peer review only - http://bmjopen.bmj.com/site/about/guidelines.xhtml

23. Dayyabu AL, Murtala Y, Grünebaum A, et al. Midwife-assisted planned home birth: an essential component of improving the safety of childbirth in Sub-Saharan Africa. *J Perinat Med*. 2018;47(1):16-21. doi:10.1515/jpm-2018-0066

24. Smith J, Banay R, Zimmerman E, Caetano V, Musheke M, Kamanga A. Barriers to provision of respectful maternity care in Zambia: results from a qualitative study through the lens of behavioral science. *BMC Pregnancy Childbirth*. 2020;20(1):26. doi:10.1186/s12884-019-2579-x

25. Biemba G, Yeboah-Antwi K, Semrau K, Hammond E, Hamer D. Who is assisting women to deliver babies within health facilities. *Anal Deliv Four Prov Zamb Austin J Public Health Epidemiol*. 2014;1(2):1007.

26. Some TD, Sombie I, Meda N. Women's perceptions of homebirths in two rural medical districts in Burkina Faso: a qualitative study. *Reprod Health*. 2011;8(1):3. doi:10.1186/1742-4755-8-3

27. Berhe R, Nigusie A. Perceptions of Home Delivery Risk and Associated Factors among Pregnant Mothers in North Achefer District, Amhara Region of Ethiopia: The Health Belief Model Perspective. 2019;(238):10.

28. Ajah LO, Onu FA, Ogbuinya OC, et al. Choice of birth place among antenatal clinic attendees in rural mission hospitals in Ebonyi State, South-East Nigeria. *PloS One*. 2019;14(11):e0211306. doi:10.1371/journal.pone.0211306

29. Lwelamira J, Safari J. Choice of Place for Childbirth: Prevalence and Determinants of Health Facility Delivery Among Women in Bahi District, Central Tanzania. *AJMS*. 2011;4.

30. Pardeshi GS, Dalvi SS, Pergulwar CR, Gite RN, Wanje SD. Trends in choosing place of delivery and assistance during delivery in Nanded district, Maharashtra, India. *J Health Popul Nutr*. 2011;29(1):71-76. doi:10.3329/jhpn.v29i1.7568

31. Scott NA, Henry EG, Kaiser JL, et al. Factors affecting home delivery among women living in remote areas of rural Zambia: a cross-sectional, mixed-methods analysis. *Int J Womens Health*. 2018;10:589-601. doi:10.2147/IJWH.S169067

32. Henry EG, Semrau K, Hamer DH, et al. The influence of quality maternity waiting homes on utilization of facilities for delivery in rural Zambia. *Reprod Health*. 2017;14(1):68. doi:10.1186/s12978-017-0328-z

33. Lori JR, Perosky J, Munro-Kramer ML, et al. Maternity waiting homes as part of a comprehensive approach to maternal and newborn care: a cross-sectional survey. *BMC Pregnancy Childbirth*. 2019;19(1):228. doi:10.1186/s12884-019-2384-6

34. Zambia Statistics Agency, Ministry of Health/Zambia, University Teaching Hospital Virology Laboratory, ICF. Demographic and Health Survey 2018. Published 2020. Accessed February 2, 2022. https://www.dhsprogram.com/pubs/pdf/FR361/FR361.pdf

For peer review only - http://bmjopen.bmj.com/site/about/guidelines.xhtml

35. Amref Health Africa. Impact of the Covid-19 Pandemic and Response on Reproductive, Maternal, Child and Adolescent Health Service Provision in Zambia. Amref Health Africa. Published 2021. Accessed December 13, 2021. https://amref.org/download/impact-of-the-covid-19-pandemic-and-response-on-reproductive-maternal-child-and-adolescent-health-service-provision-in-zambia/

36. Tiruneh GT, Karim AM, Avan BI, et al. The effect of implementation strength of basic emergency obstetric and newborn care (BEmONC) on facility deliveries and the met need for BEmONC at the primary health care level in Ethiopia. *BMC Pregnancy Childbirth*. 2018;18. doi:10.1186/s12884-018-1751-z

37. Bintabara D, Ernest A, Mpondo B. Health facility service availability and readiness to provide basic emergency obstetric and newborn care in a low-resource setting: evidence from a Tanzania National Survey. *BMJ Open*. 2019;9(2):e020608. doi:10.1136/bmjopen-2017-020608

For peer review only

STROBE Statement—Checklist of items that should be included in reports of ***cohort studies***

	Item No	Recommendation	Page No
**Title and abstract**	1	(*a*) Indicate the study's design with a commonly used term in the title or the abstract	1
		(*b*) Provide in the abstract an informative and balanced summary of what was done and what was found	1
**Introduction**			
Background/rationale	2	Explain the scientific background and rationale for the investigation being reported	1
Objectives	3	State specific objectives, including any prespecified hypotheses	1
**Methods**			
Study design	4	Present key elements of study design early in the paper	1-2
Setting	5	Describe the setting, locations, and relevant dates, including periods of recruitment, exposure, follow-up, and data collection	2-3
Participants	6	(*a*) Give the eligibility criteria, and the sources and methods of selection of participants. Describe methods of follow-up<br>(*b*) For matched studies, give matching criteria and number of exposed and unexposed	3
Variables	7	Clearly define all outcomes, exposures, predictors, potential confounders, and effect modifiers. Give diagnostic criteria, if applicable	4
Data sources/ measurement	8*	For each variable of interest, give sources of data and details of methods of assessment (measurement). Describe comparability of assessment methods if there is more than one group	4
Bias	9	Describe any efforts to address potential sources of bias	3-4
Study size	10	Explain how the study size was arrived at	3-4
Quantitative variables	11	Explain how quantitative variables were handled in the analyses. If applicable, describe which groupings were chosen and why	4-5
Statistical methods	12	(*a*) Describe all statistical methods, including those used to control for confounding<br>(*b*) Describe any methods used to examine subgroups and interactions<br>(*c*) Explain how missing data were addressed<br>(*d*) If applicable, explain how loss to follow-up was addressed<br>(*e*) Describe any sensitivity analyses	4-5
**Results**			
Participants	13*	(a) Report numbers of individuals at each stage of study—eg numbers potentially eligible, examined for eligibility, confirmed eligible, included in the study, completing follow-up, and analysed<br>(b) Give reasons for non-participation at each stage<br>(c) Consider use of a flow diagram	5
Descriptive data	14*	(a) Give characteristics of study participants (eg demographic, clinical, social) and information on exposures and potential confounders<br>(b) Indicate number of participants with missing data for each variable of interest<br>(c) Summarise follow-up time (eg, average and total amount)	5-13
Outcome data	15*	Report numbers of outcome events or summary measures over time	5

For peer review only - http://bmjopen.bmj.com/site/about/guidelines.xhtml

Main results	16	(*a*) Give unadjusted estimates and, if applicable, confounder-adjusted estimates and their precision (eg, 95% confidence interval). Make clear which confounders were adjusted for and why they were included<br>(*b*) Report category boundaries when continuous variables were categorized<br>(*c*) If relevant, consider translating estimates of relative risk into absolute risk for a meaningful time period	7-8
Other analyses	17	Report other analyses done—eg analyses of subgroups and interactions, and sensitivity analyses	n/a
**Discussion**			
Key results	18	Summarise key results with reference to study objectives	13
Limitations	19	Discuss limitations of the study, taking into account sources of potential bias or imprecision. Discuss both direction and magnitude of any potential bias	16
Interpretation	20	Give a cautious overall interpretation of results considering objectives, limitations, multiplicity of analyses, results from similar studies, and other relevant evidence	16
Generalisability	21	Discuss the generalisability (external validity) of the study results	
**Other information**			
Funding	22	Give the source of funding and the role of the funders for the present study and, if applicable, for the original study on which the present article is based	

*Give information separately for exposed and unexposed groups.

Note: An Explanation and Elaboration article discusses each checklist item and gives methodological background and published examples of transparent reporting. The STROBE checklist is best used in conjunction with this article (freely available on the Web sites of PLoS Medicine at http://www.plosmedicine.org/, Annals of Internal Medicine at http://www.annals.org/, and Epidemiology at http://www.epidem.com/). Information on the STROBE Initiative is available at http://www.strobe-statement.org.

For peer review only
